# Obesity and risk of respiratory tract infections: results of an infection-diary based cohort study

**DOI:** 10.1186/s12889-018-5172-8

**Published:** 2018-02-20

**Authors:** Livia Maccioni, Susanne Weber, Magdeldin Elgizouli, Anne-Sophie Stoehlker, Ilona Geist, Hans-Hartmut Peter, Werner Vach, Alexandra Nieters

**Affiliations:** 1 Center for Chronic Immunodeficiency (CCI), Medical Center - University of Freiburg, Faculty of Medicine, University of Freiburg, Freiburg, Germany; 2 Institute for Medical Biometry and Statistics, Faculty of Medicine and Medical Center - University of Freiburg, Freiburg, Germany

**Keywords:** Obesity, Adult respiratory tract infections, Diary, Effect modification, Life style factors

## Abstract

**Background:**

Respiratory tract infections (RTIs) are a major morbidity factor contributing largely to health care costs and individual quality of life. The aim of the study was to test whether obesity (BMI ≥ 30 kg/m^2^) is one of the risk factors underlying frequent RTIs in the German adult population.

**Methods:**

We recruited 1455 individuals between 18 to 70 years from a cross-sectional survey on airway infections in Germany and invited them to self-report in diaries incident RTIs experienced during three consecutive winter/spring seasons. RTIs reported in these 18 months and summary measures adding-up individual RTIs were the outcomes of interest.

**Results:**

Compared to individuals with normal weight, obese individuals reported a consistently higher frequency of upper and lower RTIs and predominantly fell in the upper 10% group of a diary sumscore adding-up 10 different RTI symptoms over time. Obesity was associated both with lower RTIs (_adjusted_OR = 2.02, 95%CI = 1.36–3.00) and upper RTIs (_adjusted_OR = 1.55, 95%CI = 1.22–1.96). Adjusting for demographic and lifestyle variables did only marginally affect ORs. Stratified analyses suggested a stronger association for women and effect modifications by sports activity and dietary habits.

**Conclusions:**

We confirm the association of obesity with infection burden and present evidence for putative interaction with sports activity and dietary patterns.

**Electronic supplementary material:**

The online version of this article (10.1186/s12889-018-5172-8) contains supplementary material, which is available to authorized users.

## Background

Frequent and severe respiratory tract infections (RTIs) constitute an important morbidity factor in our society and a considerable cost burden in terms of medical treatment and time of work-loss [1, 2]. RTIs are divided into upper RTIs (URTIs) including common cold, pharyngitis, otitis, sinusitis, laryngotracheitis, epiglottitis and lower RTIs (LRTIs) including bronchitis, pneumonia and bronchiolitis [3]. Individual exposure to infectious agents and host factors such as smoking [4, 5] and vitamin D status [6, 7] are believed to contribute to observed differences in RTI risk. In addition, the role of overweight (body mass index (BMI) = 25.0–29.9 kg/m^2^) and in particular obesity (BMI ≥ 30 kg/m^2^) in predisposition to RTIs is increasingly discussed [8–13]. This growing interest is driven by the rising number of overweight and obese individuals worldwide [14] and the emerging knowledge of notable immunological imbalances in association with obesity [15]. Most of the studies targeting adults explored the association of obesity with specific RTIs and their outcomes. Thus, obesity was associated with non-allergic rhinitis [8] and influenza like-illness [9]. Moreover, two population-based studies which investigated the role of obesity as risk factor for community acquired pneumonia (CAP) in the general population resulted in controversial findings [10, 11]. Two recent Danish population-based studies reported an excess of a large spectrum of RTIs including pneumonia among obese people [12, 13]. The overall aim of our study targeting the adult population in South Baden, Germany, is to identify risk factors for the susceptibility to RTIs. Here we present data on the role of obesity as contributing factor to a high RTI burden in the German society and explore effect modification by gender, sports activity and nutritional patterns.

## Methods

### Study population

Study participants (*n* = 1455) were recruited from the airway infection susceptibility (AWIS) cross sectional study querying RTI burden in an adult population in South-Baden, Germany [16]. The study protocol was approved by community officials and the Ethics Committee of the University of Freiburg (Ref. No. 258/11_120365). Based on the RTI history-score individuals of putative low, medium and high risk of future RTIs were invited to the actual sub-cohort. The RTI history score is summarizing information on the frequency and severity of RTIs and antibiotics use over the past two years, self-assessed RTI susceptibility, and occurrence of selected severe infections [16]. Study participants were requested to fill-in an additional questionnaire (baseline questionnaire) on lifestyle factors and co-morbidities and to complete monthly diaries registering the monthly occurrence and the duration (< 2 weeks, > 2 weeks) of RTIs, namely sinusitis, rhinitis, otitis media, pharyngitis/laryngitis, tonsillitis, influenza-like illness, bronchitis, pneumonia, pleurisy and other acute RTIs, from the beginning of November to the end of April of three seasons: 2012/13, 2013/14 and 2014/15. Furthermore, the intake of antibiotics, doctor visits, hospitalisation for RTIs and the impact of RTI symptoms on their daily activities were queried. Further recruitment details into the AWIS study and the present sub-cohort are presented under Additional files 1 and 2. Informed consent was obtained from all individual participants included in the study.

#### Outcome measures

In order to describe the association between obesity and RTIs, different outcome indicators were considered: outcomes **at the level of each month** [“any RTI”, “any URTI” (sinusitis, rhinitis, otitis media, pharyngitis/laryngitis and tonsillitis), “any LRTI” (bronchitis, pneumonia and pleurisy), “≥3 RTIs”, “any long lasting infection” (> 2 weeks)]; **at the level of each winter season** (“≥4 months with infections”, “≥3 long lasting infections”); and **at the individual level** (i.e. are defined once per individual and covering the overall study period). The ten specific RTI symptom categories were considered with the binary symptom indicators “infection reported” or “no infection reported” for each month. When counting the episodes for the outcome indicator “≥3 long lasting infections”, different infection symptoms were counted as separate episodes, even if they overlapped in time. However, within one symptom category at least one month without this specific infection was required to call it a new episode. We also calculated a **monthly diary RTI score**, averaging the ten RTI symptom categories with the coding “0” for “no infection reported”, “1” for “reported infection with duration < 2 weeks”, and “2” for “reported infection present with duration >2 weeks”. Missing values for individual infection items were treated as zero. If an individual RTI symptom was reported, but information on duration was missing, it was counted as “reported infection with duration < 2 weeks”. If all items were missing, no diary score was computed. The diary RTI score at the monthly level was expanded to a **score at the seasonal level** by averaging over the six months (November–April) of each season, and to an **overall score at the individual level** by averaging over all available months. The respective upper 10% of these diary scores within each month, season and overall served as additional outcome indicators.

Further variables considered in the study were age, gender, self-reported weight and height for BMI calculation (BMI was categorized as < 30 (non-obese), 25 ≤ BMI < 30 (overweight) and ≥30 (obese)), educational level, contact with children, comorbidities, removed immunological organs, smoking status, sports activity and dietary intake patterns. Details on these variables are described in the Additional file 1 and supplementary information on dietary intake patterns is presented in Additional file 3.

#### Statistical analysis

Statistical analysis was performed using Stata (version 14 STATSCorp, USA). **Descriptive statistics**: Monthly prevalences of individual RTI symptoms were computed by taking the average over all subjects available at each month and then averaging over all 18 months covered. Prevalences at the seasonal level were computed accordingly averaging over all three seasons covered. The corresponding confidence intervals (CIs) and *p*-values are based on a generalised linear model with identity link and binomial type variance together with robust variance estimates. The frequency of long lasting infections among all months with infections was analysed accordingly. However, due to the limited number of cases for tonsillitis and otitis media we determined the monthly frequency of long-lasting infections by pooling the data over all seasons and for pneumonia by pooling all indicated months.

#### Odds ratios (ORs) for outcome variables and adjustments

At the monthly level ORs were computed using a logistic regression model with a random intercept applied to the individual data for each month taking the 18 months as a categorical covariate into account in addition to the obesity status indicator. Due to its small prevalence, pleurisy was not considered as single outcome in these analyses. Outcomes at the seasonal level were analysed accordingly with the individual data for each winter season and taking into account the three seasons as a categorical covariate. Outcomes at the individual level were analysed using a logistic regression model. Results are ORs and 95% CIs. Adjusted ORs are based on including age groups and education as simultaneous categorical covariates. Furthermore, in order to study the stability of the obesity-RTI association with respect to potential confounders, ORs were adjusted by respective variables. Subjects with incomplete covariate data were excluded from multivariate analyses.

#### Subgroup analysis

Effect modification by a binary variable was assessed by fitting an overall model with the corresponding interactions parametrized so that we could directly read off the two subgroup-specific ORs. Effect modification by sports activity and nutrition patterns was explored among those representing the lower and upper third of respective scores.

## Results

### Characteristics of the study population

The study population comprised 1455 individuals (931 female and 524 male) with a median age of 51.08 years. Based on BMI calculated from self-reported weight and height, 2.1% of the population was underweight (BMI < 18.5 kg/m^2^), 54% had a normal weight (18.5 kg/m^2^ ≤ BMI < 25 kg/m^2^), 31.1% was overweight, and 12.8% was considered obese (Table 1). In women, the distribution was 2.8%, 60.21%, 25.0%, and 12.1% and in men 0.76%, 43.1%, 41.8%, and 14.3%, respectively. The study participants were mainly of medium and high educational level, non- or ex-smokers, moderately affected by selected co-morbidities and they reported rather infrequent contact to small children. Further information on the study population and completed diaries is reported in Table 1 and Additional file 4.

**Table 1:** Characteristics of the study population

		All (*N* = 1455):		Male (*N* = 524):		Female (*N* = 931):		
Variable	Category	Absolute number	Relative frequency (%)	Absolute number	Relative frequency (%)	Absolute number	Relative frequency (%)	*P*-value^d^ (gender)
BMI	< 18.5	30	2.1	4	0.76	26	2.8	< 0.001
	18.5–25	786	54.0	226	43.1	560	60.1	
	25–30	452	31.1	219	41.8	233	25.0	
	30–35	135	9.3	58	11.1	77	8.3	
	35–40	34	2.3	9	1.7	25	2.7	
	≥40	18	1.2	8	1.5	10	1.1	
	missing	0		0		0		
Age (years)	< 30	140	9.6	24	4.6	116	12.5	< 0.001
	30–40	170	11.7	39	7.4	131	14.1	
	40–50	367	25.2	110	21.0	257	27.6	
	50–60	403	27.7	162	30.9	241	25.9	
	≥60	375	25.8	189	36.1	186	20.0	
Educational level	none	4	0.28	1	0.19	3	0.32	< 0.001
	Hauptschule^a^	287	19.8	141	27.0	146	15.8	
	Realschule/Mittlere Reife^b^	470	32.4	122	23.3	348	37.5	
	Abitur^c^	261	18.0	66	12.6	195	21.0	
	university degree	428	29.5	193	36.9	235	25.4	
	missing	5		1		4		
Smoking status	never	789	54.3	248	47.4	541	58.1	< 0.001
	former smoker	461	10.0	190	39.0	271	9.1	
	current smoker	204	31.7	85	36.3	119	29.1	
	missing	1		1		0		
Contact with children	never	162	11.2	73	14.0	89	9.6	< 0.001
	rarely	574	39.5	236	45.1	338	36.4	
	weekly	292	20.1	90	17.2	202	21.7	
	daily	424	29.2	124	23.7	300	32.3	
	missing	3		1		2		
Co-morbidities:								
COPD/Lung emphysema	yes	35	2.4	23	4.4	12	1.3	< 0.001
	missing	10		3		7		
Asthma	yes	89	6.2	32	6.1	57	6.2	1.000
	missing	14		3		11		
Renal disease	yes	16	1.1	9	1.7	7	0.76	0.116
	missing	13		3		10		
Blood disease	yes	21	1.5	7	1.4	14	1.5	1.000
	missing	13		6		7		
Liver disease	yes	55	3.8	27	5.2	28	3.0	0.046
	missing	13		2		11		
Rheumatoid disease	yes	52	3.6	14	2.7	38	4.1	0.187
	missing	14		6		8		
Chronic intestinal disease	yes	45	3.1	19	3.6	26	2.8	0.431
	missing	11		3		8		
Diabetes mellitus	yes	46	3.2	23	4.4	23	2.5	0.060
	missing	13		4		9		

^a^Secondary general school, represents 9 years of school education; ^b^Intermediate secondary school, represents 10 years of school education;^c^General Higher Education Entrance Qualification, represents 12–13 years of school education; ^d^ the *p*-value for the gender difference is based on the Fisher’s exact test for comorbidities and on chi2 otherwise

### Reported RTIs over 18 months covering three winter seasons

Missing rates of single items in the returned diaries were limited and ranged from 1.2% for rhinitis and pharyngitis/laryngitis to 2.6% for other acute respiratory infections. Study participants reported most frequently rhinitis (26.6%), followed by influenza-like illness (11.4%) and pharyngitis/laryngitis (10.5%), whereas pleurisy (0.10%) was rarely experienced. Any URTI (31.5%) was more frequent than any LRTI (7.9%). Apart from the LRTIs bronchitis, pneumonia and pleurisy, which more men than women reported, all other RTIs were more prevalent among women (Table 2). Seasonal patterns of reported infections show a February peak for two of the three assessed infection seasons (2012/13 and 2014/15, see Additional file 5). Respiratory infections with a high fraction of long duration were almost exclusively LRTIs, namely pneumonia (59%), followed by bronchitis (48.2%). Men were overrepresented among those with long-lasting RTIs (Table 2).

**Table 2:** a) Prevalence of respiratory tract infections (RTIs) outcomes and b) frequency of long lasting RTIs

a)							
	All (*N* = 1455):		Male (*N* = 524):		Female (*N* = 931):		
Outcome indicators	Prevalence (%)	95% CI	Prevalence (%)	95% CI	Prevalence (%)	95% CI	*P*-value^a^ (gender)
Monthly level:							
AnyRTI	36.3	(34.9;37.7)	35.1	(32.7;37.5)	37.0	(35.2;38.7)	0.223
AnyURTI	31.5	(30.2;32.9)	29.9	(27.6;32.1)	32.4	(30.7;34.1)	0.077
AnyLRTI	7.9	(7.1; 8.8)	9.0	(7.3;10.6)	7.4	(6.4; 8.3)	0.097
Sinusitis	7.0	(6.2; 7.8)	5.3	(4.1; 6.5)	7.9	(6.9; 8.9)	< 0.001
Rhinitis	26.6	(25.4;27.9)	25.8	(23.7;27.9)	27.0	(25.4;28.6)	0.368
Otitis media	0.94	(0.67; 1.21)	0.87	(0.49; 1.24)	0.98	(0.61; 1.35)	0.674
Pharyngitis/Laryngitis	10.5	(9.6;11.3)	9.7	(8.2;11.2)	10.9	(9.8;11.9)	0.218
Tonsillitis	1.9	(1.6; 2.3)	1.4	(0.8; 2.0)	2.2	(1.8; 2.7)	0.040
Influenza-like illness	11.4	(10.6;12.1)	11.3	(10.1;12.6)	11.4	(10.4;12.4)	0.942
Bronchitis	7.8	(7.0; 8.7)	8.9	(7.2;10.5)	7.3	(6.3; 8.2)	0.102
Pneumonia	0.21	(0.11; 0.30)	0.26	(0.07; 0.45)	0.17	(0.08; 0.27)	0.433
Pleurisy	0.10	(0.03; 0.17)	0.17	(0.00; 0.34)	0.06	(0.01; 0.11)	0.220
Other acute resp. Infections	2.4	(2.0; 2.8)	1.9	(1.1; 2.7)	2.6	(2.1; 3.1)	0.137
≥ 3 RTIs	8.6	(7.8; 9.4)	8.1	(6.8; 9.4)	8.9	(7.9; 9.8)	0.362
Long RTIs	13.0	(11.9;14.0)	12.7	(10.8;14.6)	13.1	(11.8;14.4)	0.737
Upper 10% in diary score	10.0	(9.1;10.9)	10.0	(8.4;11.6)	10.0	(8.9;11.1)	0.992
Seasonal level:							
≥ 4 months RTIs	19.3	(17.6;21.0)	18.6	(15.9;21.4)	19.6	(17.5;21.8)	0.566
≥ 3 long RTIs	9.2	(8.1;10.4)	9.9	(7.8;11.9)	8.9	(7.4;10.3)	0.445
Upper 10% in diary score	10.2	(8.9;11.5)	10.7	(8.3;13.1)	9.9	(8.3;11.5)	0.602
Individual-level:							
Upper 10% in diary score	10.0	(8.4;11.5)	9.9	(7.4;12.5)	10.0	(8.1;11.9)	0.968
b)							
	All (*N* = 1455):		Male (*N* = 524):		Female (*N* = 931):		
	Frequency^b^ (%)	95% CI	Frequency^b^ (%)	95% CI	Frequency^b^ (%)	95% CI	*P*-value^a^ (gender)
Outcome indicators							
Any long RTI	35.5	(33.4;37.6)	36.2	(32.4;39.9)	35.2	(32.7;37.7)	0.674
Sinusitis	41.1	(36.7;45.5)	45.0	(35.4;54.6)	39.6	(34.8;44.5)	0.326
Rhinitis	26.2	(24.0;28.4)	27.4	(23.4;31.4)	25.5	(22.9;28.1)	0.435
Otitis media	32.6	(22.7;42.6)	36.7	(18.1;55.4)	31.1	(19.1;43.0)	0.616
Pharyngitis/Laryngitis	27.8	(24.6;30.9)	32.6	(26.7;38.6)	25.5	(22.0;29.1)	0.043
Tonsillitis	16.7	(11.7;21.8)	23.7	(12.3;35.2)	14.4	(8.9;20.0)	0.153
Influenza-like illness	26.0	(23.1;28.8)	28.8	(23.8;33.7)	24.5	(21.1;28.0)	0.175
Bronchitis	48.2	(44.5;51.9)	48.3	(41.7;54.9)	48.1	(44.0;52.2)	0.965
Pneumonia	59.0	(42.0;75.9)	66.7	(44.4;88.9)	52.4	(29.4;75.4)	0.382
Other acute resp. infections	46.5	(39.7;53.3)	55.8	(42.5;69.0)	42.9	(35.5;50.4)	0.097

^a^the *p*-value for the gender difference is based on the Fisher’s exact test for comorbidities and on chi2 otherwise

^b^for all months in which a respective infection was reported

### Association between obesity and reported RTIs

Compared to normal weight individuals, overweight and obese people consistently had a higher prevalence (Table 3) for the single RTIs, URTIs, LRTIs, as well as the other outcome parameters we looked at with other acute infections and pneumonia as the exceptions. For pneumonia, only obese subjects had a higher prevalence. The overweight group was typically falling in between the groups with normal weight and obesity (Table 3). The strongest association was seen for pneumonia and bronchitis, and accordingly, any LRTI was more strongly associated with obesity than any URTI. Long-lasting RTIs, frequent RTIs and high diary scores were also more strongly associated with obesity than the individual symptoms. Adjustments by age and education did only marginally change these estimates. Among subjects with an infection, long lasting infections were again associated with obesity, reaching significance for any RTI, rhinitis, pharyngitis/laryngitis, influenza-like illness, and bronchitis (Table 3).

**Table 3:** a) Associations of obesity with RTIs and b) with long lasting RTIs

a)							
	Prevalence (%)			(Obese vs non-obese)		(Obese vs non-obese)	
Outcome indicators	BMI < 25 (*N* = 816)	Overweight (*N* = 452)	Obese (*N* = 187)	Crude OR	95% CI	Adjusted^a^ OR	95% CI
Monthly level:							
Any RTI	33.2	39.0	43.5	1.48	(1.18; 1.85)	1.49	(1.18; 1.87)
Any URTI	28.9	33.6	38.4	1.48	(1.17; 1.87)	1.55	(1.22; 1.96)
Any LRTI	6.0	9.8	12.1	2.54	(1.69; 3.80)	2.02	(1.36; 3.00)
Sinusitis	5.7	7.9	10.6	1.99	(1.29; 3.08)	2.12	(1.36; 3.31)
Rhinitis	24.2	28.4	32.8	1.43	(1.13; 1.80)	1.53	(1.21; 1.94)
Otitis media	0.68	1.18	1.49	2.22	(0.90; 5.47)	2.31	(0.95; 5.63)
Pharyngitis/Laryngitis	9.3	11.3	13.5	1.69	(1.23; 2.33)	1.70	(1.23; 2.36)
Tonsillitis	1.7	2.3	2.1	1.36	(0.67; 2.79)	1.56	(0.77; 3.16)
Influenza-like illness	9.8	12.7	15.2	1.58	(1.23; 2.03)	1.58	(1.23; 2.03)
Bronchitis	5.9	9.8	11.7	2.38	(1.58; 3.59)	1.89	(1.26; 2.83)
Pneumonia	0.19	0.13	0.45	6.06	(1.35;27.21)	6.01	(1.30;27.90)
Other acute resp. infections	2.1	2.9	2.0	0.80	(0.41; 1.57)	0.73	(0.37; 1.43)
≥ 3 RTIs	6.8	10.2	12.8	2.15	(1.52; 3.03)	2.12	(1.50; 3.00)
Long RTIs	9.9	15.4	20.4	2.41	(1.72; 3.39)	2.14	(1.52; 3.02)
Upper 10% in diaryscore	7.5	12.2	15.7	2.21	(1.57; 3.12)	2.09	(1.48; 2.96)
Seasonal level:							
≥ 4 months RTIs	15.5	22.4	28.4	2.69	(1.62; 4.45)	2.54	(1.53; 4.21)
≥ 3 long RTIs	6.7	11.0	17.4	3.13	(2.01; 4.88)	2.81	(1.79; 4.40)
Upper 10% in diary score	6.3	13.4	19.2	4.85	(2.53; 9.32)	3.95	(2.08; 7.51)
Individual level:							
Upper 10% in diary score	6.0	13.7	18.2	2.32	(1.52; 3.52)	1.97	(1.28; 3.04)
b)							
	Frequency^b^ (%)			(Obese vs non-obese)		(Obese vs non-obese)	
Outcome indicators	BMI < 25 (*N* = 816)	Overweight (*N* = 452)	Obese (*N* = 187)	Crude OR	95% CI	Adjusted^a^ OR	95% CI
Any long RTIs	29.9	39.1	46.6	2.24	(1.64; 3.05)	1.93	(1.42; 2.63)
Sinusitis	35.9	44.3	41.8	1.77	(0.94; 3.31)	1.51	(0.80; 2.86)
Rhinitis	22.1	28.1	35.6	1.84	(1.29; 2.62)	1.71	(1.20; 2.44)
Otitis media	31.1	34.1	36.9	4.12	(0.38;45.18)	2.87	(0.26;31.54)
Pharyngitis/Laryngitis	21.9	31.8	37.4	2.42	(1.48; 3.97)	2.15	(1.32; 3.51)
Tonsillitis	16.2	14.7	22.5	3.21	(0.64;16.15)	2.98	(0.59;15.05)
Influenza-like illness	21.6	28.2	34.4	2.13	(1.34; 3.38)	1.86	(1.18; 2.94)
Bronchitis	44.0	47.5	59.8	2.08	(1.33; 3.24)	2.06	(1.32; 3.23)
Pneumonia	52.4	57.1	72.7	4.18	(0.25;81.73)	3.40	(0.17;68.52)
Other acute resp. infections	44.6	47.6	53.3	2.42	(0.58;10.14)	2.09	(0.51; 8.56)

^a^adjusted by age (continuous) and educational status (three categories)

^b^for all months in which a respective infection was reported

### Robustness of associations to confounding

For a better understanding of the robustness of the relationship between RTI burden and obesity, the effect of adjusting for putative confounders was explored (Additional file 6). The studied demographic and lifestyle variables (age, gender, education level, smoking status, contact to children, asthma, sports activity, dietary patterns and previous removal of immune organs) did only marginally affect ORs. However, adjustment for asthma, *chronic obstructive pulmonary disease* (COPD) or a summary score covering all queried co-morbidities weakened the relationship between obesity and all outcomes considerably. Adjustment for vitamin D levels among those for which serum was available (*n* = 508), had only a slight effect on the magnitude of the association between obesity and RTI outcomes.

### Effect modification by gender, sports activity and nutritional pattern

The association between obesity and RTI outcomes was more prominent for women than for men and reached statistical significance only for the former (Table 4). For most outcomes this interaction was not significant, with the individual level diary score as an exception. When looking at sports activity, for most outcomes the association with obesity was confined to those physically more active and not seen for those reporting little sports activity (Table 5). For all outcomes the association was less pronounced in the latter group (compare the ratios of ORs in Table 5), a difference that reached significance for all outcomes except those with low prevalence. Typically the prevalence of an outcome was only increased in the small group of people with obesity and higher sports activity whereas all other groups presented rather similar patterns. Similarly, the prevalence of outcomes was increased among people with obesity and a more favourable nutritional pattern, but comparable among the other groups (Table 6). The interaction reaches significance for the majority of outcomes.

**Table 4:** Association of obesity with RTIs in females and males

		Male (*N* = 524)				Female (*N* = 931)					
		Prevalence (%)				Prevalence (%)					
Outcome indicators	Approach	Non-obese (*N* = 449)	Obese (*N* = 75)	OR	95% CI	Non-obese (*N* = 819)	Obese (*N* = 112)	OR	95% CI	OR male/OR female	*P*-value
Monthly level:											
Any RTI	crude	34.4	39.3	1.24	(0.86; 1.79)	35.7	45.9	1.66	(1.24; 2.22)	0.75	0.221
	adjusted^a^			1.23	(0.86; 1.78)			1.67	(1.25; 2.23)	0.74	0.196
Any URTI	crude	29.2	33.9	1.18	(0.80; 1.73)	31.2	41.1	1.72	(1.27; 2.31)	0.69	0.129
	adjusted^a^			1.22	(0.84; 1.79)			1.79	(1.33; 2.41)	0.68	0.121
Any LRTI	crude	8.5	11.7	1.97	(1.02; 3.78)	6.7	12.1	2.92	(1.75; 4.87)	0.67	0.351
	adjusted^a^			1.47	(0.78; 2.80)			2.43	(1.47; 4.03)	0.60	0.225
Sinusitis	crude	4.8	8.1	1.51	(0.69; 3.29)	7.3	12.1	2.36	(1.38; 4.01)	0.64	0.353
	adjusted^a^			1.55	(0.71; 3.40)			2.48	(1.45; 4.25)	0.63	0.331
Rhinitis	crude	25.3	29.3	1.10	(0.76; 1.61)	25.9	35.0	1.68	(1.25; 2.26)	0.66	0.089
	adjusted^a^			1.19	(0.82; 1.73)			1.79	(1.33; 2.40)	0.66	0.091
Otitis media	crude	0.85	0.92	0.60	(0.11; 3.19)	0.86	1.81	3.89	(1.34;11.24)	0.15	0.066
	adjusted^a^			0.62	(0.12; 3.20)			4.20	(1.47;12.02)	0.15	0.054
Pharyngitis/Laryngitis	crude	9.4	11.4	1.54	(0.91; 2.61)	10.3	14.8	1.82	(1.22; 2.73)	0.84	0.616
	adjusted^a^			1.50	(0.88; 2.55)			1.84	(1.22; 2.77)	0.81	0.542
Tonsillitis	crude	1.49	0.79	0.56	(0.12; 2.55)	2.11	2.93	1.82	(0.83; 4.00)	0.31	0.177
	adjusted^a^			0.66	(0.15; 2.98)			1.99	(0.90; 4.37)	0.33	0.205
Influenza-like illness	crude	10.8	14.6	1.35	(0.90; 2.03)	10.8	15.4	1.73	(1.26; 2.38)	0.78	0.347
	adjusted^a^			1.38	(0.92; 2.07)			1.72	(1.25; 2.36)	0.81	0.406
Bronchitis	crude	8.5	11.3	1.77	(0.91; 3.46)	6.6	11.9	2.83	(1.69; 4.76)	0.63	0.277
	adjusted^a^			1.32	(0.68; 2.55)			2.35	(1.41; 3.92)	0.56	0.172
Pneumonia	crude	0.24	0.40	2.30	(0.22;23.53)	0.13	0.46	10.94	(1.47;81.47)	0.21	0.316
	adjusted^a^			2.62	(0.24;28.13)			9.92	(1.34;73.33)	0.26	0.391
Other acute resp. infections	crude	2.12	0.59	0.15	(0.03; 0.76)	2.55	2.90	1.29	(0.62; 2.72)	0.11	0.018
	adjusted^a^			0.13	(0.03; 0.68)			1.18	(0.56; 2.50)	0.11	0.017
≥ 3 RTIs	crude	7.6	10.7	1.44	(0.81; 2.58)	8.2	13.9	2.68	(1.74; 4.13)	0.54	0.093
	adjusted^a^			1.37	(0.77; 2.45)			2.70	(1.75; 4.15)	0.51	0.066
Long RTIs	crude	11.9	18.0	2.05	(1.18; 3.59)	11.9	21.8	2.69	(1.74; 4.15)	0.76	0.454
	adjusted^a^			1.72	(0.98; 2.99)			2.47	(1.60; 3.81)	0.69	0.309
Upper 10% in diary score	crude	9.4	13.9	1.49	(0.84; 2.65)	9.1	16.7	2.78	(1.81; 4.27)	0.54	0.089
	adjusted^a^			1.35	(0.76; 2.39)			2.69	(1.75; 4.14)	0.50	0.057
Seasonal level:											
≥ 4 months RTIs	crude	17.6	25.0	2.26	(1.00; 5.09)	18.1	30.3	2.99	(1.58; 5.66)	0.76	0.592
	adjusted^a^			1.99	(0.88; 4.46)			2.94	(1.56; 5.56)	0.68	0.450
≥ 3 long RTIs	crude	8.2	15.8	2.57	(1.24; 5.34)	8.3	18.3	3.51	(2.02; 6.09)	0.73	0.502
	adjusted^a^			2.24	(1.07; 4.70)			3.20	(1.83; 5.60)	0.70	0.449
Upper 10% in diary score	crude	9.7	16.8	2.45	(0.82; 7.31)	8.4	20.6	7.13	(3.15;16.12)	0.34	0.124
	adjusted^a^			1.89	(0.64; 5.57)			5.95	(2.67;13.26)	0.32	0.093
Individual level:											
Upper 10% in diary score	crude	9.8	10.7	1.10	(0.50; 2.44)	8.2	23.2	3.39	(2.05; 5.62)	0.32	0.019
	adjusted^a^			0.90	(0.40; 2.03)			2.95	(1.76; 4.95)	0.31	0.015

^a^adjusted by age (continuous) and educational status (three categories)

**Table 5:** Effect modification by sports activity

		Less active (lower third, *N* = 485)				High active (upper third, *N* = 488)					
		Prevalence (%)				Prevalence (%)					
Outcome indicators	Approach	Non-obese (*N* = 379)	Obese (*N* = 106)	OR	95% CI	Non-obese (*N* = 454)	Obese (*N* = 34)	OR	95% CI	OR less/OR more active	*P*-value
Monthly level:											
Any RTI	crude	37.9	38.8	0.98	(0.72; 1.35)	33.7	51.6	2.56	(1.54; 4.26)	0.38	0.002
	adjusted^a^			1.00	(0.73; 1.37)			2.58	(1.56; 4.27)	0.39	0.002
Any URTI	crude	32.0	33.8	1.03	(0.75; 1.42)	29.2	46.7	2.63	(1.56; 4.43)	0.39	0.003
	adjusted^a^			1.08	(0.78; 1.49)			2.70	(1.61; 4.52)	0.40	0.003
Any LRTI	crude	10.3	10.3	1.19	(0.67; 2.13)	6.1	14.6	5.17	(2.14;12.49)	0.23	0.006
Sinusitis	adjusted^a^			0.94	(0.53; 1.67)			4.31	(1.82;10.21)	0.22	0.004
	crude	6.9	9.3	1.36	(0.72; 2.58)	6.1	15.1	4.35	(1.70;11.13)	0.31	0.045
	adjusted^a^			1.42	(0.75; 2.70)			4.16	(1.62;10.69)	0.34	0.063
Rhinitis	crude	27.1	29.0	1.02	(0.74; 1.41)	24.2	39.3	2.46	(1.47; 4.09)	0.42	0.004
	adjusted^a^			1.09	(0.79; 1.50)			2.52	(1.52; 4.16)	0.43	0.005
Otitis media	crude	0.84	0.71	1.03	(0.28; 3.75)	0.84	1.60	1.69	(0.28;10.22)	0.61	0.659
	adjusted^a^			0.97	(0.27; 3.45)			1.80	(0.32;10.02)	0.54	0.570
Pharyngitis/Laryngitis	crude	10.1	10.4	1.15	(0.73; 1.81)	10.2	22.8	3.98	(2.03; 7.80)	0.29	0.003
	adjusted^a^			1.14	(0.72; 1.80)			3.85	(1.96; 7.57)	0.30	0.003
Tonsillitis	crude	1.9	1.8	1.07	(0.37; 3.13)	2.1	3.9	4.25	(1.24;14.61)	0.25	0.098
	adjusted^a^			1.28	(0.45; 3.60)			5.33	(1.56;18.15)	0.24	0.079
Influenza-like illness	crude	12.0	13.6	1.08	(0.77; 1.52)	10.4	22.6	3.45	(2.08; 5.75)	0.31	< 0.001
	adjusted^a^			1.08	(0.77; 1.52)			3.46	(2.08; 5.75)	0.31	< 0.001
Bronchitis	crude	10.3	10.0	1.09	(0.60; 1.97)	6.0	14.2	4.78	(1.94;11.78)	0.23	0.007
	adjusted^a^			0.86	(0.48; 1.54)			3.93	(1.62; 9.51)	0.22	0.005
Pneumonia	crude	0.18	0.43	5.07	(0.64;40.15)	0.18	0.95	27.64	(1.02;751.94)	0.18	0.372
	adjusted^a^			5.02	(0.56;44.91)			19.80	(0.95;410.73)	0.25	0.449
Other acute resp. infections	crude	3.1	1.8	0.48	(0.18; 1.32)	1.9	2.2	1.53	(0.37; 6.41)	0.31	0.196
	adjusted^a^			0.44	(0.16; 1.21)			1.33	(0.32; 5.46)	0.33	0.214
≥ 3 RTIs	crude	9.0	10.6	1.26	(0.78; 2.05)	7.5	19.9	5.57	(2.73;11.34)	0.23	< 0.001
	adjusted^a^			1.20	(0.74; 1.94)			5.07	(2.50;10.27)	0.24	< 0.001
Long RTIs	crude	14.9	16.5	1.19	(0.73; 1.93)	10.4	26.4	5.37	(2.53;11.40)	0.22	0.001
	adjusted^a^			1.07	(0.66; 1.75)			4.91	(2.31;10.43)	0.22	< 0.001
Upper 10% in diary score	crude	11.1	13.3	1.21	(0.74; 1.96)	8.2	21.9	5.53	(2.65;11.52)	0.22	< 0.001
	adjusted^a^			1.13	(0.70; 1.84)			5.10	(2.45;10.61)	0.22	< 0.001
Seasonal level:											
≥ 4 months RTIs	crude	21.1	24.3	1.32	(0.66; 2.64)	14.8	32.8	6.27	(2.07;18.95)	0.21	0.019
	adjusted^a^			1.30	(0.65; 2.61)			5.86	(1.94;17.69)	0.22	0.023
≥ 3 long RTIs	crude	10.2	13.2	1.59	(0.82; 3.12)	7.6	26.0	7.54	(2.88;19.69)	0.21	0.009
	adjusted^a^			1.35	(0.69; 2.65)			6.59	(2.53;17.16)	0.20	0.007
Upper 10% in diary score	crude	10.9	14.5	1.63	(0.64; 4.16)	7.1	33.7	39.36	(8.94;173.29)	0.04	< 0.001
	adjusted^a^			1.27	(0.50; 3.23)			31.05	(7.52;128.22)	0.04	< 0.001
Individual level:											
Upper 10% in diary score	crude	12.1	13.2	1.10	(0.58; 2.09)	6.6	35.3	7.71	(3.48;17.07)	0.14	< 0.001
	adjusted^a^			0.94	(0.49; 1.81)			7.00	(3.12;15.75)	0.13	< 0.001

^a^adjusted by age (continuous) and educational status (three categories)

**Table 6:** Effect modification by nutritional status

		More unfavourable nutrition (lower third, *N* = 379)				More favourable nutrition (upper third, *N* = 530)					
		Prevalence (%)				Prevalence (%)					
Outcome indicators	Approach	Non-obese (*N* = 325)	Obese (*N* = 54)	OR	95% CI	Non-obese (*N* = 467)	Obese (*N* = 63)	OR	95% CI	OR unfavourable/OR favourable nutrition	*P*-value
Monthly level:											
Any RTI	crude	34.8	37.4	1.10	(0.72; 1.68)	35.8	49.3	1.99	(1.34; 2.94)	0.55	0.045
	adjusted^a^			1.13	(0.74; 1.74)			2.02	(1.36; 2.99)	0.56	0.049
Any URTI	crude	30.0	32.9	1.13	(0.73; 1.77)	31.0	43.8	2.01	(1.34; 3.01)	0.57	0.064
	adjusted^a^			1.22	(0.78; 1.91)			2.10	(1.40; 3.14)	0.58	0.075
Any LRTI	crude	7.5	8.4	1.75	(0.79; 3.85)	8.0	14.5	3.13	(1.58; 6.19)	0.56	0.274
	adjusted^a^			1.22	(0.56; 2.67)			2.43	(1.24; 4.74)	0.50	0.187
Sinusitis	crude	5.9	7.7	1.54	(0.64; 3.67)	7.2	14.2	2.40	(1.14; 5.04)	0.64	0.443
	adjusted^a^			1.55	(0.64; 3.75)			2.33	(1.09; 4.94)	0.67	0.491
Rhinitis	crude	26.7	28.3	1.04	(0.67; 1.62)	25.5	37.5	1.99	(1.33; 2.97)	0.52	0.034
	adjusted^a^			1.17	(0.75; 1.82)			2.14	(1.43; 3.19)	0.55	0.044
Otitis media	crude	1.35	0.60	0.46	(0.06; 3.43)	0.82	1.16	1.73	(0.36; 8.27)	0.27	0.312
	adjusted^a^			0.61	(0.08; 4.63)			2.17	(0.48; 9.84)	0.28	0.320
Pharyngitis/Laryngitis	crude	8.6	8.5	1.10	(0.58; 2.09)	10.3	18.3	2.80	(1.65; 4.77)	0.39	0.028
	adjusted^a^			1.08	(0.57; 2.06)			2.79	(1.63; 4.76)	0.39	0.025
Tonsillitis	crude	2.25	0.46	0.14	(0.02; 1.04)	1.72	2.09	1.63	(0.47; 5.66)	0.09	0.042
	adjusted^a^			0.19	(0.03; 1.40)			1.99	(0.57; 6.95)	0.10	0.050
Influenza-like illness	crude	11.9	13.9	1.22	(0.77; 1.95)	10.4	20.3	2.43	(1.60; 3.70)	0.50	0.032
	adjusted^a^			1.23	(0.77; 1.98)			2.42	(1.58; 3.71)	0.51	0.035
Bronchitis	crude	7.5	8.4	1.77	(0.80; 3.94)	7.9	13.8	2.71	(1.34; 5.45)	0.65	0.434
	adjusted^a^			1.23	(0.55; 2.70)			2.09	(1.05; 4.17)	0.58	0.312
Pneumonia	crude	0.06	0.00	1.00	(.;.)	0.25	0.99	14.92	(1.10;202.01)	0.07	0.042
	adjusted^a^			1.00	(.;.)			7.30	(0.89;59.69)	0.14	0.064
Other acute resp. infections	crude	2.5	1.4	0.73	(0.19; 2.75)	2.1	3.3	1.38	(0.46; 4.13)	0.53	0.464
	adjusted^a^			0.64	(0.17; 2.43)			1.22	(0.41; 3.61)	0.52	0.456
≥ 3 RTIs	crude	8.6	7.9	1.01	(0.51; 2.00)	8.0	17.3	3.72	(2.11; 6.55)	0.27	0.004
	adjusted^a^			1.01	(0.51; 2.00)			3.53	(2.00; 6.25)	0.29	0.005
Long RTIs	crude	11.4	14.8	1.77	(0.89; 3.50)	12.0	26.4	3.87	(2.12; 7.05)	0.46	0.091
	adjusted^a^			1.50	(0.76; 2.99)			3.31	(1.81; 6.05)	0.46	0.088
Upper 10% in diary score	crude	9.6	9.9	1.26	(0.64; 2.47)	9.3	21.3	3.58	(2.01; 6.36)	0.35	0.021
	adjusted^a^			1.14	(0.58; 2.24)			3.24	(1.82; 5.78)	0.35	0.020
Seasonal level:											
≥ 4 months RTIs	crude	18.5	19.8	1.11	(0.41; 3.05)	18.1	38.5	6.31	(2.59;15.41)	0.18	0.012
	adjusted^a^			1.10	(0.40; 3.02)			5.82	(2.39;14.18)	0.19	0.014
≥ 3 long RTIs	crude	8.0	10.7	1.64	(0.63; 4.32)	8.8	24.5	5.52	(2.55;11.95)	0.30	0.052
	adjusted^a^			1.38	(0.51; 3.73)			4.72	(2.15;10.34)	0.29	0.053
Upper 10% in diary score	crude	9.5	9.1	0.89	(0.18; 4.36)	8.9	28.7	14.73	(4.41;49.21)	0.06	0.006
	adjusted^a^			0.61	(0.12; 3.02)			10.65	(3.41;33.28)	0.06	0.004
Individual level:											
Upper 10% in diary score	crude	9.5	9.3	0.97	(0.36; 2.61)	8.3	25.4	3.74	(1.94; 7.19)	0.26	0.026
	adjusted^a^			0.77	(0.28; 2.10)			3.12	(1.58; 6.15)	0.25	0.022

^a^adjusted by age (continuous) and educational status (three categories)

## Discussion

RTIs constitute an important morbidity factor considering the high health care costs, the time lost from work, and the impaired quality of life among those recurrently affected [1, 2, 17]. Obesity belongs to one of the host risk factors for RTI and has possibly an emerging role due to the dramatically increasing prevalence of obesity worldwide. In the present study, we report on the association of obesity with individual RTIs as well as with a diary score summarising different incident RTI symptoms over a period of 18 months. Our investigation could demonstrate an association between obesity and RTIs confirming previous findings on influenza-like illness [9], bronchitis [18] and pneumonia [10, 12]. We also saw an association between obesity and rhinitis, sinusitis and pharyngitis/laryngitis. An elevated risk for sinusitis among obese was also reported in a population-based cohort of Danish women [13]. None of the two Danish population-based studies [12, 13] used ORs of monthly prevalence, but hazard ratios (HRs), as they could identify events on a daily basis. The HR of 1.6 [12] for the association with RTIs and the HR of 1.48 [13] for the association with URTIs are, however, of similar magnitude to the risk estimates which we observed. Mechanistically, excess adiposity might weigh down host defence as several mouse as well as human studies have suggested [19, 20]. The here observed associations were more prominent for LRTIs compared to URTIs, but evident for both, and more pronounced when considering long lasting or frequent RTIs compared to single symptoms. Based on the infection diary data, we generated a RTI diary score summing-up all ten symptoms and allowing to average per month, per whole season or over the whole period of three years. Considering the upper ten percentile of the distribution of such scores as an outcome, associations were typically stronger than when considering single symptoms, and interactions were more pronounced. Moreover, the results of the seasonal score were very similar or even stronger than those of the three-years score, arguing for the adequacy to query six months infectious events in future studies to identify the infection-prone sub-group of the population.

Lifestyle habits seem to contribute to an individual’s risk for RTI. Among them, cigarette smoking has been reported as a major environmental risk factor for recurrent and severe RTIs [4, 5]. Frequent contact to small children [21, 22], vitamin D deficiency [23, 24], and lack of physical activity [25, 26] constitute other exposures associated with heightened RTI risks. Moreover, higher levels of education were associated with a lower risk of CAP [27]. Based on those previous findings we investigated their role as possible confounders. The association between obesity and RTIs remained nearly unchanged after adjustment for age, gender, educational status, contact to children, smoking status, sports activity and nutrition scores, suggesting that the association is not markedly confounded by the effects of these factors on both BMI and the risk of infections. Also additional adjustment by measured serum vitamin D in a subgroup for which measurements were available did not change the risk estimates considerably. This supports arguments that the observed associations between obesity and RTI burden are due to physiological differences in the immune responsiveness between obese and non-obese individuals rather than lifestyle differences. In addition, some chronic diseases, foremost asthma and COPD, are associated with both an increased risk of RTIs and obesity [28–32]. Considering these associations we investigated the effect of asthma, COPD and a co-morbidity score – summarizing the other chronic conditions – on the relationship between obesity and individual RTIs and the RTI diary score. Adjusting for these conditions individually and even more so in a combined fashion resulted in a considerable attenuation of the association between obesity and considered RTI outcomes. Hence part of the association between infections and obesity might be explainable by associations of co-morbidities with both.

We see a gender difference in the observed associations with more noticeable findings for women. A significantly increased risk for combined RTIs was also restricted to women in a Danish blood donor cohort [12]. Several lines of research support this notion: Szabova et al. and Ilavska et al. reported gender-dependent effects of obesity on the immune system [33, 34]. The effect of BMI on a variety of immune parameters including those with relevance for immune defence was much more apparent in women than in men [34]. NK cells (CD3-/CD16+/CD56+), represent first-line cells for the clearing of virus-infected cells. Reduced levels of these cells reported for obese women, but not for respective men, might underlie the gender effect seen in our study.

We also investigated a potential effect modification by sports activity and nutrition. Interestingly, an association between obesity and RTIs was evident only for those obese individuals who reported a higher level of sports activity. Thus, only the group of obese people who engaged in more intensive sports activity reported RTIs more frequently whereas obese people with low sports activity and non-obese with low or high sports activity showed comparable lower prevalences for most outcomes. We hypothesize that oxidative stress induced by vigorous aerobic as well as anaerobic sports activity is exacerbated in people with obesity, but not in normal weight individuals. Evidence supporting this has been previously published [35]. An imbalanced oxidative stress status may have negative consequences on mounting an appropriate immune response towards respiratory pathogens. Excessive reactive oxygen species (ROS) was shown to hinder T cell responses to viral infection [36] and ROS accumulation was detected in autophagy-deficient effector T cells rendering them incapable of controlling viral infections [37].

A similar surprising result was found when studying the effect modification by dietary patterns. Here we queried the participants’ dietary habits and classified them as adhering to a more favourable or more unfavourable dietary pattern according to Winkler et al. [38]. Aware of the limitations of a one-time assessment of a habitual diet, we found a more pronounced relationship between obesity and infections among obese people who reported an apparent healthier diet. Thus, again only the group of obese individuals who presumably eat a healthier diet showed an increased risk of RTIs. The question arises as to whether misreporting of dietary habits among these individuals with and without RTIs may explain the puzzle. One can imagine that obese individuals may have an increased perception of RTI related symptoms experiencing the contradiction between living a healthy lifestyle and being affected by excess weight and frequent infections. On the other hand the inconspicuous results from the non-obese population with respect to favourable and unfavourable diet pattern would somewhat argue against this explanation. Alternatively, among the group of people with obesity a genetically defined subgroup may exist predisposing to both, excess body weight and proneness to infections.

### Strengths and limitations

As strengths of our study we count 1) its sample size, allowing for the analysis of effect modification, 2) its prospective design involving 18 months infection diaries for the exploration of the relationship between BMI and subsequent RTI frequency and severity, 3) the comprehensive information on lifestyle and co-morbidities allowing to study the interplay of such factors on their effect on infections, and 4) the wide range of outcome indicators considered. The uniformity of the results with respect to these outcomes also suggests that in the field of airway infection morbidity, studies may be comparable despite the fact that they often concentrate on different RTI outcomes. In line with the majority of epidemiological studies in this area of research, our study suffers from some limitations, including the reliance on self-reported outcomes and exposure data with the risk of misclassification. However, we found - for instance - a good agreement between BMI derived from self-reported weight and height data and BMI calculated from measured values available for a sub-cohort (*n* = 508). Moreover, differential misclassification which would substantially bias the relationship between obesity and RTIs is rather unexpected in this setting. The disproportional selection of women into the study may negatively impact the generalizability of some of our results.

## Conclusions

In conclusion, in this prospective cohort of adults we found obese overrepresented among those reporting frequent and long-lasting RTIs. In line with previous epidemiological studies as well as basic research data we observed a stronger effect of obesity on infection risk for women compared to men. The interesting interaction with sports activity and presumed nutrition awaits follow-up investigations in subsequent studies that ideally shall provide improved measurements of the entire spectrum of physical activity and dietary habits.

## Additional files

Additional file 1: Details into the AWIS study and the sub-cohort. (DOCX 20 kb)

Additional file 2: Flow diagram describing the study population. (DOCX 27 kb)

Additional file 3: Development of the nutrition score to assess more favourable and unfavourable dietary pattern. (DOCX 112 kb)

Additional file 4: Distribution of the number of diaries and months available per subject. (DOCX 14 kb)

Additional file 5: Seasonal prevalence patterns for each symptom indicator. (DOCX 61 kb)

Additional file 6: Association of obesity with RTIs adjusted by age, gender, education level, smoking, contact to children, asthma, COPD, Co-morbidity, physical activity, nutrition, removed organs and vitamin D^a^. (DOCX 39 kb)

## Abbreviations

95%CI: 95% confidence interval
AWIS: Airwayinfection susceptibility
BMI: Body mass index
CAP: Community acquired pneumonia
COPD: Chronic obstructive pulmonary disease
HRs: Hazard ratios
LRTI: Lower RTI
ORs: Odds ratios
ROS: Reactive oxygen species
RTI: Respiratory tract infection
URTI: Upper RTI
