# Supplementary material for: Obesity and risk of respiratory tract infections: results of an infection-diary based cohort study
Source: BMC Public Health. 2018 Feb 20;18:271. doi: 10.1186/s12889-018-5172-8 (PMC5819164; doi:10.1186/s12889-018-5172-8)
Supplement: Supplementary file 1 — Details into the AWIS study and the sub-cohort. (DOCX 20 kb) [file 12889_2018_5172_MOESM1_ESM.docx]

Additional file 1: details into the AWIS study and the sub-cohort

Background information on the Airway Infection Susceptibility (AWIS) cross-sectional study

Recruitment into the AWIS study of individuals residing in South Baden, Germany (regions of Breisgau/Hochschwarzwald/Freiburg and Loerrach) was started in December 2011. The study protocol was approved by community officials and the Ethics Committee of the University of Freiburg (Ref. No. 258/11_120365). A questionnaire (background questionnaire) asking for the frequency and severity of individual RTI symptoms (sinusitis, rhinitis, otitis media, pharyngitis/laryngitis, tonsillitis, influenza-like illness, bronchitis, pneumonia, pleurisy and other acute respiratory infections), prescription of antibiotics, infection-related hospitalizations, occurrence of selected severe infections and self-assessed proneness to RTI in addition to demographic information, co-morbidities, smoking behaviour, contact to small children, was mailed to 70.000 randomly selected individuals in the age range of 18-70 years. Overall, 12.908 individuals completed the questionnaire (Additional file 2) and agreed to participate in the study (response rate of 18.4%). The response rate was higher in women (21.2%) than in men (15.7%). Informed consent was obtained from all individual participants included in the study.

Participation in the infection diary sub-cohort

The diaries were distributed and returned in three months periods between November 2012 and April 2015. This subsampling was stratified by the RTI history score into participants of putative low risk (≤4, n=1330), medium risk (>4 and <17, n=9914) and high risk (history score ≥ 17, n=1664). Within these three groups 79.62% (1059/1330), 32.36% (3208/9914) and 80.59% (1341/1664) were invited to participate, 39.09% (414/1059), 33.39% (1071/3208) and 43.4% (582/1341) of these accepted the invitation. 88.65%, 74.4% and 79.9% of the latter filled in at least one diary, and among these 92.37%, 90.46% and 84.95% completed infection diaries for at least 4 months and reported their weight and height for BMI calculation (Additional file 4).

Further variables considered

From the background questionnaire, age, gender, self-reported weight and height for BMI calculation, educational level (No school leaving certificate, “Hauptschule”, Intermediate school leaving certificate, General qualification for university entrance, University degree), contact with children (never, rarely, weekly, daily), comorbidities (yes, no for chronic obstructive pulmonary disease (COPD)/lung emphysema, asthma, renal disease, blood disease, liver disease, rheumatoid disease, chronic intestinal disease and diabetes mellitus), and removed organs (tonsils, nasal polyps, appendix, spleen, thymus), was taken. A comorbidity score was calculated by counting the comorbidities present. A score for removed organs was calculated analogously. BMI was categorized as <30 (not obese) and ≥30 (obese). Overweight was defined as 25≤BMI<30. Age of participants was calculated as difference of study entry date and birth date. Age was subdivided in five categories (<30, 30-40, 40-50, 50-60, ≥ 60 years). Smoking status (no, current smoker, former smoker) and sports activity were assessed in the baseline questionnaire. Sports activity was queried in 14 items (walking/strolling, hiking/Nordic walking, running, cycling, gym (lift weights), gym (aerobics), dancing, cross-country skiing, alpine skiing/ snowboarding, ball sports (like soccer, handball, volleyball), swimming, horse-back riding, yoga/pilates/Thai Chi, other sports) in four categories (never, 1-3 times per month, 1-3 times per week, >3 times per week). These items were summarized in a sports activity score representing the calorie consumption implied by the combined reported sports activities per month for a person with a body weight of 70 kg and assuming 30 minutes duration of any reported activity. For calculation of the calorie consumption the *Metabolic Equivalent of Task* (1MET=1kcal/kg/hour) for the respective activities according to Ainsworth et al. was assigned [[1](#_ENREF_1)]. The values representing 30 minutes of activity were then multiplied with the factors 0, 2, 8, and 16 for the four frequency categories (never, 1-3 times per month, 1-3 times per week, more than 3 times per week), respectively. Missing values were considered as zero. Concerning “other” sport activities, the calorie consumption was estimated as an average over climbing, golf and inline skating, the most frequently mentioned activities in a free text question. The activity score is the sum over all items and reflects the total calorie consumption per month. A higher score implies a higher level of sports activity.

Information on dietary patterns was captured by requesting information on the frequency of intake of 15 food groups (meat and sausages, venison/poultry, fish, eggs, milk and milk products, cereals, noodles, potatoes, rice, soja/pulse, cake/sweets and snacks, cooked vegetables, salad and raw vegetables, fruits and juices, and instant meal/fast food). The participants filled-in whether they consume these groups never, once, twice, three times, or more than three times per day/week/month. These consumption frequencies were translated into six categories, namely never, once per month or less, several times per month, once per week, several times per week, and almost daily. Similar to Winkler et al.[[2](#_ENREF_2)] and based on the consumption recommendations of the German Nutrition Society “DGE-Ernährungskreis” [[3](#_ENREF_3), [4](#_ENREF_4)], respective consumption frequencies for each nutrition item were assigned to one of three values, “2” for a “favourable consumption”, “1” for” normal consumption” and “0” for an “unfavourable consumption” frequency. The nutrition score covers the sum of all queried food items (Additional file 3). A higher score reflects a more favourable dietary pattern.

1. Ainsworth BE, Haskell WL, Herrmann SD, Meckes N, Bassett DR, Jr., Tudor-Locke C, Greer JL, Vezina J, Whitt-Glover MC, Leon AS: 2011 Compendium of Physical Activities: a second update of codes and MET values. Medicine and science in sports and exercise 2011, 43(8):1575-1581.

2. Winkler G, Schwertner B, Döring A: Kurzmethoden zur Charakterisierung des Ernährungsmusters: Einsatz und Auswertung eines Food-Frequency-Fragebogens. Ernährungsumschau 1995, 42:289-291.

3. von Ruesten A, Oberritter H: The German Food Pyramid. In: Diet Quality: An Evidence-Based Approach, Volume 2. edn. Edited by Preedy RV, Hunter L-A, Patel BV. New York, NY: Springer New York; 2013: 153-165.

4. DGE. DGE-Ernährungskreis. https://[www.dge.de/ernaehrungspraxis/vollwertige-ernaehrung/ernaehrungskreis/](http://www.dge.de/ernaehrungspraxis/vollwertige-ernaehrung/ernaehrungskreis/). Accessed 8 Sept 2016.
