# Supplementary material for: Obesity and risk of respiratory tract infections: results of an infection-diary based cohort study
Source: BMC Public Health. 2018 Feb 20;18:271. doi: 10.1186/s12889-018-5172-8 (PMC5819164; doi:10.1186/s12889-018-5172-8)
Supplement: Supplementary file 2 — Flow diagram describing the study population. (DOCX 27 kb) [file 12889_2018_5172_MOESM2_ESM.docx]

Additional file 2: Flow diagram describing the study population
