# Supplementary material for: Obesity and risk of respiratory tract infections: results of an infection-diary based cohort study
Source: BMC Public Health. 2018 Feb 20;18:271. doi: 10.1186/s12889-018-5172-8 (PMC5819164; doi:10.1186/s12889-018-5172-8)
Supplement: Supplementary file 3 — Development of the nutrition score to assess more favourable and unfavourable dietary pattern. (DOCX 112 kb) [file 12889_2018_5172_MOESM3_ESM.docx]

Additional file 3: Development of the nutrition score to assess more favourable and unfavourable dietary pattern

| **Categories used by Winkler G, Schwertner B, Döring A. „Kurzmethoden zur Charakterisierung des Ernährungsmusters: Einsatz und Auswertung eines Food-Frequency-Fragebogens“. Ernährungsumschau. 1995;42:289-91** | | | | | | |  | |  | |  |  |  |  |
| --- | --- | --- | --- | --- | --- | --- | --- | --- | --- | --- | --- | --- | --- | --- |
|  | |  | | |  | | |  | |  |  |  |  |  |
| 1 | | almost daily | | | | | |  | |  |  |  |  |  |
| 2 | | several times per week | | | | | |  | |  |  |  |  |  |
| 3 | | about once per week | | | | | |  | |  |  |  |  |  |
| 4 | | several times per month | | | | | |  | |  |  |  |  |  |
| 5 | | once or less per month | | | | | |  | |  |  |  |  |  |
| 6 | | never | | | | | |  | |  |  |  |  |  |
|  | |  | | |  | | |  | |  |  |  |  |  |
| **Indexing by Winkler:** | |  | | |  | | |  | |  |  |  |  |  |
| optimal consumption frequency ("optimale Verzehrshäufigkeit") | | 2 | | |  | | |  | |  |  |  |  |  |
| normal consumption frequency ("normale Verzehrshäufigkeit") | | 1 | | |  | | |  | |  |  |  |  |  |
| deviating consumption frequency ("abweichende Verzehrshäufigkeit") | | 0 | | |  | | |  | |  |  |  |  |  |
|  | |  | | |  | | |  | |  |  |  |  |  |
|  | | |  | | |  |  | |  | |  |  |  |  |
| Two variables capture the consumption of each food item: one variable (`var') for the frequency, one variable (`var'_unit) for the unit (per day, week, month) | | | | | | | | | | | | | | |
|  | | |  | | |  |  | |  | |  |  |  |  |
| **possible responses in baseline questionnaire** | | | | | | |  | |  | |  |  |  |  |
| **(-> colors indicate grouping according to the six categories as in Winkler et al.)** | | | | | | | | | | | |  |  |  |
| Possible combination of the two variables indicating the frequency (`var') and the unit (`var'_unit) of the several food items | | | | | | | | | | | | |  |  |
| (displayed are the combination of the values assigned according to the questionnaire (`var';`var'_unit)) | | | | | | | | | | | |  |  |  |
|  |  | | |  | | |  | |  | |  |  |  |  |
|  | |  | | |  | | |  | |  |  |  |  |  |
|  | `var' | | | `var'_unit | | | | | | |  |  |  |  |
|  | | never | | | per day | | | per week | | per month |  |  |  |  |
|  | | once | | |  | | |  | |  |  |  |  |  |
|  |  | twice | | |  | | |  | |  |  |  |  |  |
|  |  | three times | | |  | | |  | |  |  |  |  |  |
|  |  | more than three times | | |  | | |  | |  |  |  |  |  |

| **Food-Items captured in baseline questionnaire and comparison with considered food items in Winkler et al.:** | | | | | | |  |
| --- | --- | --- | --- | --- | --- | --- | --- |
|  |  |  |  |  |  |  |  |
| **Items in baseline questionnaire** | | *Was this Item considered by Winkler et al.?:* | category considered as "optimal" in Winkler et al. | | category according to the German Nutrition Society (consumption recommendations of the “DGE-Ernährungskreis” (reference 19)) | |  |
| meat and sausages | | *yes; as two categories, but valued equally* | 3&4 | (about once per week & several times per month) |  | (1 dose per week) |  |
| venison/poultry | | *no* | - |  |  | (1 dose per week) |  |
| fish | | *yes* | 2&3 | (several times per week & about once per week) |  | (1 dose per week) |  |
| eggs | | *yes* | 3-6 | (about once per week or less) |  | (up to 3 doses per week) |  |
| milk and milk products | | *no* | - |  |  | (daily) |  |
| cereals | | *two categories valued differently: (whole-grain-,brown-,and crisp bread) & (oat flakes, muesli, cornflakes)* | (1) or (1&2) resp. | (almost daily & several times per week) |  | (daily) |  |
| noodles | | *yes; pasta ("Teigwaren")* | 1 | (almost daily) |  | (daily) |  |
| potatoes | | *yes* | 1 | (almost daily) |  | (daily) |  |
| rice | | *yes* | 1 | (almost daily) |  | (daily) |  |
| soja/pulse | | *no* | - |  |  | (daily) (assigned to vegetable category) |  |
| cake/sweets and snacks | | *two categories valued equally: (cake etc.) & (salty pastries)* | 5&6 | (once or less per month & never) | - | - |  |
| cooked vegetables | | *yes* | 1 | (almost daily) | 2 | (daily) |  |
| salad and raw vegetables | | *yes* | 1 | (almost daily) | 2 | (daily) |  |
| fruits and juices | | *fresh fruit (category category "fruit juices" left out)* | 1 | (almost daily) | 3 | (daily) |  |
| instant meal/fast food | | *no* | - |  | - | - |  |
|  |  |  |  |  |  |  |  |
|  |  |  |  |  |  |  |  |
|  |  |  |  |  |  |  |  |

|  |  | **Assigned Values for nutrition score (0=unfavourable consumption, 1=normal consumption, 2=favourable consumption)** | | | | | |  |
| --- | --- | --- | --- | --- | --- | --- | --- | --- |
|  |  |  |  |  |  |  |  |  |
|  |  | almost daily | several times per week | about once per week | several times per month | once or les per month | never |  |
|  | category | 1 | 2 | 3 | 4 | 5 | 6 |  |
| (combination of `var' and `var'_unit:) | | (2-5;1) | (3-5;2) | (2;2) & (5;3) | (3-4;3) | (2;3) | `var'=1 | as in Winkler et al? |
| meat and sausages | | 0 | 1 | 2 | 2 | 1 | 0 | yes |
| venison/poultry | | 0 | 1 | 2 | 2 | 1 | 0 | yes |
| fish | | 1 | 2 | 2 | 1 | 0 | 0 | yes |
| eggs | | 1 | 2 | 1 | 0 | 0 | 0 | no |
| milk and milk products | | 2 | 1 | 0 | 0 | 0 | 0 | - |
| cereals | | 2 | 2 | 1 | 0 | 0 | 0 | Winkler: as for oat flakes (except category 4) |
| noodles | | 2 | 1 | 0 | 0 | 0 | 0 | yes |
| potatoes | | 2 | 1 | 0 | 0 | 0 | 0 | yes |
| rice | | 2 | 1 | 0 | 0 | 0 | 0 | yes |
| soja/pulse | | 2 | 1 | 0 | 0 | 0 | 0 | - |
| cake/sweets and snacks | | 0 | 0 | 1 | 1 | 2 | 2 | yes |
| cooked vegetables | | 2 | 1 | 0 | 0 | 0 | 0 | yes |
| salad and raw vegetables | | 2 | 1 | 0 | 0 | 0 | 0 | yes |
| fruits and juices | | 2 | 1 | 1 | 0 | 0 | 0 | yes |
| instant meal/fast food | | 0 | 0 | 0 | 0 | 1 | 2 | - |

Additional file 4: distribution of the number of diaries and months available per subject

| **Months** | **Starting year** | | | **Total** |
| --- | --- | --- | --- | --- |
|  | **2012/2013** | **2013/2014** | **2014/2015** |  |
| **4** | 0 | 2 | 0 | 2 |
| **5** | 15 | 8 | 4 | 27 |
| **6** | 107 | 90 | 43 | 240 |
| **7** | 4 | 3 | 0 | 7 |
| **8** | 8 | 4 | 0 | 12 |
| **9** | 68 | 76 | 0 | 144 |
| **10** | 3 | 3 | 0 | 6 |
| **11** | 12 | 21 | 0 | 33 |
| **12** | 117 | 300 | 0 | 417 |
| **13** | 4 | 0 | 0 | 4 |
| **14** | 13 | 0 | 0 | 13 |
| **15** | 120 | 0 | 0 | 120 |
| **16** | 7 | 0 | 0 | 7 |
| **17** | 24 | 0 | 0 | 24 |
| **18** | 399 | 0 | 0 | 399 |
| **Total** | 901 | 507 | 47 | 1455 |

Additional file 5: Seasonal prevalence patterns for each symptom indicator


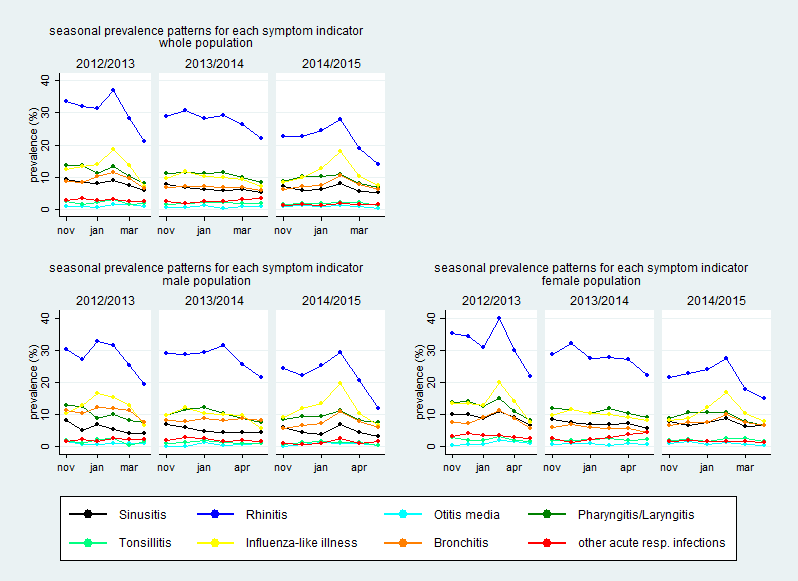


Additional file 6: Association of obesity with RTIs adjusted by age, gender, education level, smoking, contact to children, asthma, COPD, Co-morbidity, physical activity, nutrition, removed organs and vitamin D^a^

|  |  | **Adjusted^b^** |  |  |  |  |  |
| --- | --- | --- | --- | --- | --- | --- | --- |
|  | **Crude** | **Age** | **Gender** | **Education** | **Smoking** | **Contact to children** | **Asthma** |
| **Outcome indicators** | **OR / 95% CI** | **OR / 95% CI** | **OR / 95% CI** | **OR / 95% CI** | **OR / 95% CI** | **OR / 95% CI** | **OR / 95% CI** |
| **Monthly level** |  |  |  |  |  |  |  |
| **Any RTI** | 1.48 | 1.55 | 1.48 | 1.45 | 1.45 | 1.50 | 1.34 |
|  | ( 1.18; 1.85) | ( 1.23; 1.95) | ( 1.18; 1.86) | ( 1.15; 1.82) | ( 1.15; 1.82) | ( 1.19; 1.88) | ( 1.07; 1.68) |
| **Any URTI** | 1.48 | 1.60 | 1.49 | 1.48 | 1.46 | 1.51 | 1.34 |
|  | ( 1.17; 1.87) | ( 1.27; 2.02) | ( 1.17; 1.88) | ( 1.17; 1.88) | ( 1.15; 1.85) | ( 1.19; 1.91) | ( 1.06; 1.70) |
| **Any LRTI** | 2.54 | 2.17 | 2.52 | 2.25 | 2.42 | 2.54 | 2.09 |
|  | ( 1.69; 3.80) | ( 1.45; 3.23) | ( 1.69; 3.77) | ( 1.50; 3.35) | ( 1.62; 3.63) | ( 1.70; 3.79) | ( 1.40; 3.11) |
| **Sinusitis** | 1.99 | 2.10 | 2.03 | 2.10 | 1.93 | 2.00 | 1.84 |
|  | ( 1.29; 3.08) | ( 1.35; 3.27) | ( 1.31; 3.15) | ( 1.35; 3.26) | ( 1.25; 2.99) | ( 1.29; 3.10) | ( 1.18; 2.86) |
| **Rhinitis** | 1.43 | 1.57 | 1.43 | 1.45 | 1.41 | 1.47 | 1.32 |
|  | ( 1.13; 1.80) | ( 1.25; 1.98) | ( 1.13; 1.81) | ( 1.14; 1.83) | ( 1.11; 1.78) | ( 1.16; 1.85) | ( 1.05; 1.66) |
| **Otitis media** | 2.22 | 2.44 | 2.22 | 2.23 | 2.18 | 2.12 | 1.93 |
|  | ( 0.90; 5.47) | ( 0.99; 6.04) | ( 0.90; 5.46) | ( 0.91; 5.50) | ( 0.88; 5.38) | ( 0.86; 5.23) | ( 0.81; 4.60) |
| **Pharyngitis/Laryngitis** | 1.69 | 1.76 | 1.71 | 1.69 | 1.66 | 1.72 | 1.51 |
|  | ( 1.23; 2.33) | ( 1.27; 2.43) | ( 1.24; 2.36) | ( 1.22; 2.34) | ( 1.21; 2.30) | ( 1.25; 2.37) | ( 1.09; 2.08) |
| **Tonsillitis** | 1.36 | 1.65 | 1.37 | 1.35 | 1.38 | 1.35 | 1.14 |
|  | ( 0.67; 2.79) | ( 0.82; 3.33) | ( 0.67; 2.79) | ( 0.66; 2.79) | ( 0.68; 2.83) | ( 0.66; 2.78) | ( 0.56; 2.33) |
| **Influenza-like illness** | 1.58 | 1.67 | 1.58 | 1.51 | 1.55 | 1.60 | 1.47 |
|  | ( 1.23; 2.03) | ( 1.30; 2.15) | ( 1.23; 2.03) | ( 1.18; 1.94) | ( 1.21; 1.99) | ( 1.25; 2.05) | ( 1.15; 1.89) |
| **Bronchitis** | 2.38 | 2.02 | 2.37 | 2.12 | 2.30 | 2.38 | 1.97 |
|  | ( 1.58; 3.59) | ( 1.35; 3.03) | ( 1.58; 3.57) | ( 1.41; 3.19) | ( 1.53; 3.46) | ( 1.58; 3.58) | ( 1.31; 2.95) |
| **Pneumonia** | 6.06 | 5.82 | 6.05 | 5.55 | 5.61 | 6.18 | 4.46 |
|  | ( 1.35;27.21) | ( 1.30;26.07) | ( 1.35;27.16) | ( 1.25;24.60) | ( 1.28;24.53) | ( 1.36;28.06) | ( 1.05;19.05) |
| **Other acute resp. infections** | 0.80 | 0.78 | 0.79 | 0.75 | 0.75 | 0.79 | 0.74 |
|  | ( 0.41; 1.57) | ( 0.39; 1.53) | ( 0.40; 1.56) | ( 0.38; 1.48) | ( 0.38; 1.47) | ( 0.40; 1.54) | ( 0.38; 1.46) |
| **≥3 RTIs** | 2.15 | 2.24 | 2.15 | 2.09 | 2.10 | 2.18 | 1.86 |
|  | ( 1.52; 3.03) | ( 1.58; 3.17) | ( 1.52; 3.04) | ( 1.48; 2.96) | ( 1.49; 2.97) | ( 1.55; 3.08) | ( 1.32; 2.62) |
| **Long RTIs** | 2.41 | 2.29 | 2.43 | 2.22 | 2.32 | 2.43 | 2.06 |
|  | ( 1.72; 3.39) | ( 1.63; 3.23) | ( 1.72; 3.41) | ( 1.58; 3.13) | ( 1.65; 3.26) | ( 1.73; 3.41) | ( 1.47; 2.89) |
| **Upper 10% in diary score** | 2.21 | 2.23 | 2.22 | 2.09 | 2.14 | 2.25 | 1.89 |
|  | ( 1.57; 3.12) | ( 1.58; 3.15) | ( 1.57; 3.13) | ( 1.48; 2.96) | ( 1.52; 3.02) | ( 1.60; 3.17) | ( 1.35; 2.64) |
| **Seasonal level:** |  |  |  |  |  |  |  |
| **≥4 months RTIs** | 2.69 | 2.76 | 2.69 | 2.53 | 2.52 | 2.74 | 2.30 |
|  | ( 1.62; 4.45) | ( 1.66; 4.58) | ( 1.62; 4.46) | ( 1.53; 4.19) | ( 1.53; 4.17) | ( 1.65; 4.54) | ( 1.40; 3.77) |
| **≥3 long RTIs** | 3.13 | 2.92 | 3.14 | 3.01 | 3.04 | 3.18 | 2.65 |
|  | ( 2.01; 4.88) | ( 1.87; 4.56) | ( 2.01; 4.89) | ( 1.92; 4.71) | ( 1.95; 4.72) | ( 2.04; 4.96) | ( 1.72; 4.10) |
| **Upper 10% in diary score** | 4.85 | 4.49 | 4.85 | 4.22 | 4.40 | 5.00 | 3.55 |
|  | ( 2.53; 9.32) | ( 2.34; 8.62) | ( 2.53; 9.32) | ( 2.21; 8.05) | ( 2.31; 8.38) | ( 2.60; 9.59) | ( 1.89; 6.66) |
| **Individual level** |  |  |  |  |  |  |  |
| **Upper 10% in diary score** | 2.32 | 2.18 | 2.32 | 2.08 | 2.20 | 2.36 | 1.92 |
|  | ( 1.52; 3.52) | ( 1.43; 3.33) | ( 1.52; 3.53) | ( 1.35; 3.19) | ( 1.44; 3.36) | ( 1.55; 3.61) | ( 1.23; 3.01) |
|  | **Adjusted** |  |  |  |  |  |  |
|  | **COPD** | **All co-morbidities** | **Physical activity** | **Nutrition** | **Removed organs** | **VIT-D subgroup (N=508, obese=63) - unadj.^a^** | **VIT-D subgroup - adjusted for VIT-D^a^** |
| **Outcome indicators** | **OR / 95% CI** | **OR / 95% CI** | **OR / 95% CI** | **OR / 95% CI** | **OR / 95% CI** | **OR / 95% CI** | **OR / 95% CI** |
| **Monthly level** |  |  |  |  |  |  |  |
| **Any RTI** | 1.42 | 1.22 | 1.44 | 1.49 | 1.43 | 1.52 | 1.49 |
|  | ( 1.14; 1.78) | ( 0.97; 1.53) | ( 1.14; 1.81) | ( 1.18; 1.86) | ( 1.14; 1.79) | ( 1.02; 2.27) | ( 0.99; 2.23) |
| **Any URTI** | 1.44 | 1.23 | 1.46 | 1.49 | 1.44 | 1.50 | 1.47 |
|  | ( 1.14; 1.82) | ( 0.97; 1.55) | ( 1.15; 1.85) | ( 1.17; 1.88) | ( 1.14; 1.82) | ( 1.00; 2.26) | ( 0.98; 2.22) |
| **Any LRTI** | 2.31 | 1.61 | 2.39 | 2.56 | 2.32 | 3.17 | 3.22 |
|  | ( 1.56; 3.42) | ( 1.08; 2.40) | ( 1.59; 3.59) | ( 1.71; 3.84) | ( 1.55; 3.46) | ( 1.71; 5.89) | ( 1.73; 5.99) |
| **Sinusitis** | 2.04 | 1.57 | 2.01 | 2.02 | 1.83 | 2.25 | 2.19 |
|  | ( 1.31; 3.17) | ( 1.00; 2.45) | ( 1.29; 3.13) | ( 1.30; 3.12) | ( 1.18; 2.82) | ( 1.14; 4.46) | ( 1.10; 4.35) |
| **Rhinitis** | 1.39 | 1.22 | 1.40 | 1.43 | 1.40 | 1.65 | 1.62 |
|  | ( 1.10; 1.75) | ( 0.97; 1.55) | ( 1.11; 1.78) | ( 1.13; 1.81) | ( 1.11; 1.77) | ( 1.10; 2.46) | ( 1.09; 2.43) |
| **Otitis media** | 2.27 | 1.57 | 2.43 | 2.25 | 2.03 | 2.70 | 2.23 |
|  | ( 0.92; 5.65) | ( 0.67; 3.72) | ( 0.97; 6.11) | ( 0.91; 5.58) | ( 0.85; 4.81) | ( 0.62;11.72) | ( 0.51; 9.83) |
| **Pharyngitis/Laryngitis** | 1.64 | 1.34 | 1.74 | 1.71 | 1.62 | 1.52 | 1.50 |
|  | ( 1.19; 2.27) | ( 0.97; 1.85) | ( 1.25; 2.40) | ( 1.24; 2.35) | ( 1.18; 2.23) | ( 0.91; 2.54) | ( 0.89; 2.52) |
| **Tonsillitis** | 1.30 | 1.13 | 1.45 | 1.36 | 1.49 | 1.39 | 1.28 |
|  | ( 0.63; 2.70) | ( 0.53; 2.39) | ( 0.70; 2.99) | ( 0.67; 2.79) | ( 0.74; 3.01) | ( 0.41; 4.76) | ( 0.37; 4.38) |
| **Influenza-like illness** | 1.54 | 1.32 | 1.57 | 1.58 | 1.53 | 1.79 | 1.81 |
|  | ( 1.20; 1.98) | ( 1.03; 1.70) | ( 1.22; 2.02) | ( 1.23; 2.02) | ( 1.19; 1.96) | ( 1.20; 2.67) | ( 1.21; 2.70) |
| **Bronchitis** | 2.17 | 1.52 | 2.25 | 2.41 | 2.18 | 3.22 | 3.27 |
|  | ( 1.45; 3.24) | ( 1.01; 2.29) | ( 1.49; 3.41) | ( 1.60; 3.63) | ( 1.45; 3.29) | ( 1.72; 6.03) | ( 1.74; 6.15) |
| **Pneumonia** | 5.90 | 3.73 | 6.60 | 6.31 | 4.95 | 8.85 | 8.44 |
|  | ( 1.52;22.91) | ( 0.92;15.14) | ( 1.47;29.70) | ( 1.42;28.07) | ( 1.11;22.17) | ( 0.63;124.49) | ( 0.62;115.75) |
| **other acute resp. infections** | 0.81 | 0.64 | 0.77 | 0.80 | 0.76 | 1.04 | 1.00 |
|  | ( 0.42; 1.56) | ( 0.32; 1.26) | ( 0.39; 1.51) | ( 0.41; 1.58) | ( 0.39; 1.49) | ( 0.44; 2.43) | ( 0.43; 2.33) |
| **≥3 RTIs** | 2.05 | 1.54 | 2.16 | 2.15 | 2.01 | 2.60 | 2.61 |
|  | ( 1.45; 2.89) | ( 1.10; 2.17) | ( 1.52; 3.06) | ( 1.52; 3.04) | ( 1.43; 2.83) | ( 1.53; 4.40) | ( 1.54; 4.43) |
| **Long RTIs** | 2.31 | 1.75 | 2.29 | 2.44 | 2.28 | 2.86 | 2.73 |
|  | ( 1.65; 3.24) | ( 1.25; 2.45) | ( 1.62; 3.23) | ( 1.73; 3.43) | ( 1.63; 3.20) | ( 1.67; 4.88) | ( 1.60; 4.67) |
| **Upper 10% in diary score** | 2.13 | 1.53 | 2.13 | 2.23 | 2.08 | 2.93 | 2.86 |
|  | ( 1.52; 2.99) | ( 1.10; 2.14) | ( 1.51; 3.02) | ( 1.58; 3.14) | ( 1.48; 2.92) | ( 1.72; 4.98) | ( 1.68; 4.86) |
| **Seasonal level** |  |  |  |  |  |  |  |
| **≥4 months RTIs** | 2.43 | 1.84 | 2.39 | 2.71 | 2.46 | 3.44 | 3.37 |
|  | ( 1.48; 3.97) | ( 1.12; 3.03) | ( 1.44; 3.97) | ( 1.64; 4.49) | ( 1.50; 4.06) | ( 1.57; 7.53) | ( 1.54; 7.37) |
| **≥3 long RTIs** | 3.10 | 2.27 | 3.09 | 3.16 | 2.94 | 3.11 | 2.97 |
|  | ( 2.00; 4.81) | ( 1.47; 3.52) | ( 1.97; 4.84) | ( 2.03; 4.91) | ( 1.90; 4.57) | ( 1.65; 5.86) | ( 1.58; 5.59) |
| **Upper 10% in diary score** | 4.49 | 2.47 | 4.61 | 4.90 | 4.43 | 4.10 | 3.96 |
|  | ( 2.38; 8.49) | ( 1.33; 4.61) | ( 2.38; 8.92) | ( 2.55; 9.42) | ( 2.31; 8.46) | ( 1.85; 9.08) | ( 1.79; 8.78) |
| **Individual level** |  |  |  |  |  |  |  |
| **Upper 10% in diary score** | 2.22 | 1.48 | 2.17 | 2.33 | 2.17 | 3.15 | 3.05 |
|  | ( 1.44; 3.43) | ( 0.93; 2.36) | ( 1.42; 3.34) | ( 1.53; 3.55) | ( 1.42; 3.32) | ( 1.59; 6.24) | ( 1.54; 6.07) |

^a^ to allow for a comprehensive identification of biomarkers and other features of people susceptible to RTI, AWIS participants were invited into the study center for a detailed interview, anthropometric measurements, lung function test, pulse oximetry, a general medical examination as well as the collection of biosamples (blood, urine, oral and nasal swabs) and measurement of selected biomarkers including serum vitamin D levels. The recruitment started in August 2014 and it is still ongoing. From 1455 invited AWIS participants (546 male and 903 female), 550 (200 male and 348 female) were recruited until the end of January 2016. 508 of these (186 male and 322 female) with available BMI were included in the present analysis

b Adjustment was performed separately for each factor. Gender, education, smoking, contact to small children, asthma, co-morbidity score, score of removed organs, were added as categorical covariates; age groups, sport activity score, nutrition score, serum vitamin D were added as continuous covariates.
