# Supplementary material for: Obesity and risk of respiratory tract infections: results of an infection-diary based cohort study
Source: BMC Public Health. 2018 Feb 20;18:271. doi: 10.1186/s12889-018-5172-8 (PMC5819164; doi:10.1186/s12889-018-5172-8)
Supplement: Supplementary file 4 — Distribution of the number of diaries and months available per subject. (DOCX 14 kb) [file 12889_2018_5172_MOESM4_ESM.docx]

Additional file 4: distribution of the number of diaries and months available per subject

| **Months** | **Starting year** | | | **Total** |
| --- | --- | --- | --- | --- |
|  | **2012/2013** | **2013/2014** | **2014/2015** |  |
| **4** | 0 | 2 | 0 | 2 |
| **5** | 15 | 8 | 4 | 27 |
| **6** | 107 | 90 | 43 | 240 |
| **7** | 4 | 3 | 0 | 7 |
| **8** | 8 | 4 | 0 | 12 |
| **9** | 68 | 76 | 0 | 144 |
| **10** | 3 | 3 | 0 | 6 |
| **11** | 12 | 21 | 0 | 33 |
| **12** | 117 | 300 | 0 | 417 |
| **13** | 4 | 0 | 0 | 4 |
| **14** | 13 | 0 | 0 | 13 |
| **15** | 120 | 0 | 0 | 120 |
| **16** | 7 | 0 | 0 | 7 |
| **17** | 24 | 0 | 0 | 24 |
| **18** | 399 | 0 | 0 | 399 |
| **Total** | 901 | 507 | 47 | 1455 |
