# Supplementary figures and images for: Obesity and risk of respiratory tract infections: results of an infection-diary based cohort study
Source: BMC Public Health. 2018 Feb 20;18:271. doi: 10.1186/s12889-018-5172-8 (PMC5819164; doi:10.1186/s12889-018-5172-8)

Additional file 5: Seasonal prevalence patterns for each symptom indicator


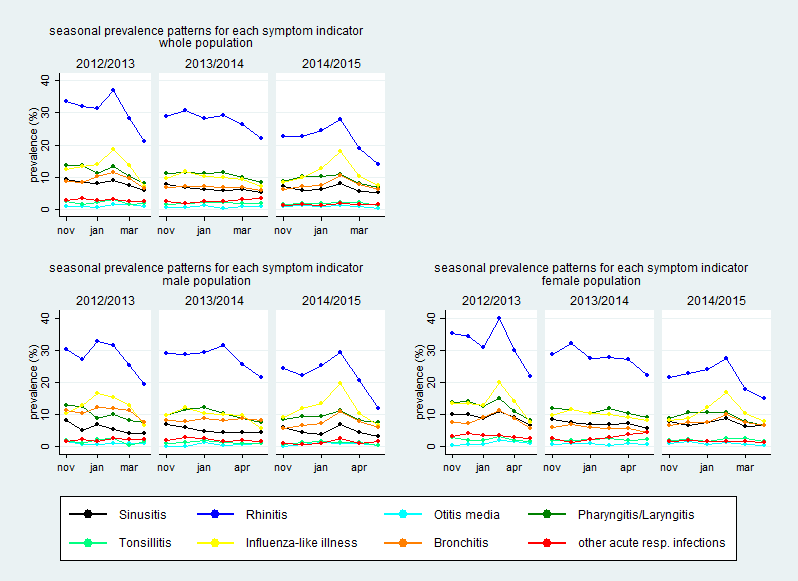

Supplement: Supplementary file 5 — Seasonal prevalence patterns for each symptom indicator. (DOCX 61 kb) [file 12889_2018_5172_MOESM5_ESM.docx]
