# Supplementary material for: Obesity and risk of respiratory tract infections: results of an infection-diary based cohort study
Source: BMC Public Health. 2018 Feb 20;18:271. doi: 10.1186/s12889-018-5172-8 (PMC5819164; doi:10.1186/s12889-018-5172-8)
Supplement: Supplementary file 6 — Association of obesity with RTIs adjusted by age, gender, education level, smoking, contact to children, asthma, COPD, Co-morbidity, physical activity, nutrition, removed organs and vitamin Da. (DOCX 39 kb) [file 12889_2018_5172_MOESM6_ESM.docx]

Additional file 6: Association of obesity with RTIs adjusted by age, gender, education level, smoking, contact to children, asthma, COPD, Co-morbidity, physical activity, nutrition, removed organs and vitamin D^a^

|  |  | **Adjusted^b^** |  |  |  |  |  |
| --- | --- | --- | --- | --- | --- | --- | --- |
|  | **Crude** | **Age** | **Gender** | **Education** | **Smoking** | **Contact to children** | **Asthma** |
| **Outcome indicators** | **OR / 95% CI** | **OR / 95% CI** | **OR / 95% CI** | **OR / 95% CI** | **OR / 95% CI** | **OR / 95% CI** | **OR / 95% CI** |
| **Monthly level** |  |  |  |  |  |  |  |
| **Any RTI** | 1.48 | 1.55 | 1.48 | 1.45 | 1.45 | 1.50 | 1.34 |
|  | ( 1.18; 1.85) | ( 1.23; 1.95) | ( 1.18; 1.86) | ( 1.15; 1.82) | ( 1.15; 1.82) | ( 1.19; 1.88) | ( 1.07; 1.68) |
| **Any URTI** | 1.48 | 1.60 | 1.49 | 1.48 | 1.46 | 1.51 | 1.34 |
|  | ( 1.17; 1.87) | ( 1.27; 2.02) | ( 1.17; 1.88) | ( 1.17; 1.88) | ( 1.15; 1.85) | ( 1.19; 1.91) | ( 1.06; 1.70) |
| **Any LRTI** | 2.54 | 2.17 | 2.52 | 2.25 | 2.42 | 2.54 | 2.09 |
|  | ( 1.69; 3.80) | ( 1.45; 3.23) | ( 1.69; 3.77) | ( 1.50; 3.35) | ( 1.62; 3.63) | ( 1.70; 3.79) | ( 1.40; 3.11) |
| **Sinusitis** | 1.99 | 2.10 | 2.03 | 2.10 | 1.93 | 2.00 | 1.84 |
|  | ( 1.29; 3.08) | ( 1.35; 3.27) | ( 1.31; 3.15) | ( 1.35; 3.26) | ( 1.25; 2.99) | ( 1.29; 3.10) | ( 1.18; 2.86) |
| **Rhinitis** | 1.43 | 1.57 | 1.43 | 1.45 | 1.41 | 1.47 | 1.32 |
|  | ( 1.13; 1.80) | ( 1.25; 1.98) | ( 1.13; 1.81) | ( 1.14; 1.83) | ( 1.11; 1.78) | ( 1.16; 1.85) | ( 1.05; 1.66) |
| **Otitis media** | 2.22 | 2.44 | 2.22 | 2.23 | 2.18 | 2.12 | 1.93 |
|  | ( 0.90; 5.47) | ( 0.99; 6.04) | ( 0.90; 5.46) | ( 0.91; 5.50) | ( 0.88; 5.38) | ( 0.86; 5.23) | ( 0.81; 4.60) |
| **Pharyngitis/Laryngitis** | 1.69 | 1.76 | 1.71 | 1.69 | 1.66 | 1.72 | 1.51 |
|  | ( 1.23; 2.33) | ( 1.27; 2.43) | ( 1.24; 2.36) | ( 1.22; 2.34) | ( 1.21; 2.30) | ( 1.25; 2.37) | ( 1.09; 2.08) |
| **Tonsillitis** | 1.36 | 1.65 | 1.37 | 1.35 | 1.38 | 1.35 | 1.14 |
|  | ( 0.67; 2.79) | ( 0.82; 3.33) | ( 0.67; 2.79) | ( 0.66; 2.79) | ( 0.68; 2.83) | ( 0.66; 2.78) | ( 0.56; 2.33) |
| **Influenza-like illness** | 1.58 | 1.67 | 1.58 | 1.51 | 1.55 | 1.60 | 1.47 |
|  | ( 1.23; 2.03) | ( 1.30; 2.15) | ( 1.23; 2.03) | ( 1.18; 1.94) | ( 1.21; 1.99) | ( 1.25; 2.05) | ( 1.15; 1.89) |
| **Bronchitis** | 2.38 | 2.02 | 2.37 | 2.12 | 2.30 | 2.38 | 1.97 |
|  | ( 1.58; 3.59) | ( 1.35; 3.03) | ( 1.58; 3.57) | ( 1.41; 3.19) | ( 1.53; 3.46) | ( 1.58; 3.58) | ( 1.31; 2.95) |
| **Pneumonia** | 6.06 | 5.82 | 6.05 | 5.55 | 5.61 | 6.18 | 4.46 |
|  | ( 1.35;27.21) | ( 1.30;26.07) | ( 1.35;27.16) | ( 1.25;24.60) | ( 1.28;24.53) | ( 1.36;28.06) | ( 1.05;19.05) |
| **Other acute resp. infections** | 0.80 | 0.78 | 0.79 | 0.75 | 0.75 | 0.79 | 0.74 |
|  | ( 0.41; 1.57) | ( 0.39; 1.53) | ( 0.40; 1.56) | ( 0.38; 1.48) | ( 0.38; 1.47) | ( 0.40; 1.54) | ( 0.38; 1.46) |
| **≥3 RTIs** | 2.15 | 2.24 | 2.15 | 2.09 | 2.10 | 2.18 | 1.86 |
|  | ( 1.52; 3.03) | ( 1.58; 3.17) | ( 1.52; 3.04) | ( 1.48; 2.96) | ( 1.49; 2.97) | ( 1.55; 3.08) | ( 1.32; 2.62) |
| **Long RTIs** | 2.41 | 2.29 | 2.43 | 2.22 | 2.32 | 2.43 | 2.06 |
|  | ( 1.72; 3.39) | ( 1.63; 3.23) | ( 1.72; 3.41) | ( 1.58; 3.13) | ( 1.65; 3.26) | ( 1.73; 3.41) | ( 1.47; 2.89) |
| **Upper 10% in diary score** | 2.21 | 2.23 | 2.22 | 2.09 | 2.14 | 2.25 | 1.89 |
|  | ( 1.57; 3.12) | ( 1.58; 3.15) | ( 1.57; 3.13) | ( 1.48; 2.96) | ( 1.52; 3.02) | ( 1.60; 3.17) | ( 1.35; 2.64) |
| **Seasonal level:** |  |  |  |  |  |  |  |
| **≥4 months RTIs** | 2.69 | 2.76 | 2.69 | 2.53 | 2.52 | 2.74 | 2.30 |
|  | ( 1.62; 4.45) | ( 1.66; 4.58) | ( 1.62; 4.46) | ( 1.53; 4.19) | ( 1.53; 4.17) | ( 1.65; 4.54) | ( 1.40; 3.77) |
| **≥3 long RTIs** | 3.13 | 2.92 | 3.14 | 3.01 | 3.04 | 3.18 | 2.65 |
|  | ( 2.01; 4.88) | ( 1.87; 4.56) | ( 2.01; 4.89) | ( 1.92; 4.71) | ( 1.95; 4.72) | ( 2.04; 4.96) | ( 1.72; 4.10) |
| **Upper 10% in diary score** | 4.85 | 4.49 | 4.85 | 4.22 | 4.40 | 5.00 | 3.55 |
|  | ( 2.53; 9.32) | ( 2.34; 8.62) | ( 2.53; 9.32) | ( 2.21; 8.05) | ( 2.31; 8.38) | ( 2.60; 9.59) | ( 1.89; 6.66) |
| **Individual level** |  |  |  |  |  |  |  |
| **Upper 10% in diary score** | 2.32 | 2.18 | 2.32 | 2.08 | 2.20 | 2.36 | 1.92 |
|  | ( 1.52; 3.52) | ( 1.43; 3.33) | ( 1.52; 3.53) | ( 1.35; 3.19) | ( 1.44; 3.36) | ( 1.55; 3.61) | ( 1.23; 3.01) |
|  | **Adjusted** |  |  |  |  |  |  |
|  | **COPD** | **All co-morbidities** | **Physical activity** | **Nutrition** | **Removed organs** | **VIT-D subgroup (N=508, obese=63) - unadj.^a^** | **VIT-D subgroup - adjusted for VIT-D^a^** |
| **Outcome indicators** | **OR / 95% CI** | **OR / 95% CI** | **OR / 95% CI** | **OR / 95% CI** | **OR / 95% CI** | **OR / 95% CI** | **OR / 95% CI** |
| **Monthly level** |  |  |  |  |  |  |  |
| **Any RTI** | 1.42 | 1.22 | 1.44 | 1.49 | 1.43 | 1.52 | 1.49 |
|  | ( 1.14; 1.78) | ( 0.97; 1.53) | ( 1.14; 1.81) | ( 1.18; 1.86) | ( 1.14; 1.79) | ( 1.02; 2.27) | ( 0.99; 2.23) |
| **Any URTI** | 1.44 | 1.23 | 1.46 | 1.49 | 1.44 | 1.50 | 1.47 |
|  | ( 1.14; 1.82) | ( 0.97; 1.55) | ( 1.15; 1.85) | ( 1.17; 1.88) | ( 1.14; 1.82) | ( 1.00; 2.26) | ( 0.98; 2.22) |
| **Any LRTI** | 2.31 | 1.61 | 2.39 | 2.56 | 2.32 | 3.17 | 3.22 |
|  | ( 1.56; 3.42) | ( 1.08; 2.40) | ( 1.59; 3.59) | ( 1.71; 3.84) | ( 1.55; 3.46) | ( 1.71; 5.89) | ( 1.73; 5.99) |
| **Sinusitis** | 2.04 | 1.57 | 2.01 | 2.02 | 1.83 | 2.25 | 2.19 |
|  | ( 1.31; 3.17) | ( 1.00; 2.45) | ( 1.29; 3.13) | ( 1.30; 3.12) | ( 1.18; 2.82) | ( 1.14; 4.46) | ( 1.10; 4.35) |
| **Rhinitis** | 1.39 | 1.22 | 1.40 | 1.43 | 1.40 | 1.65 | 1.62 |
|  | ( 1.10; 1.75) | ( 0.97; 1.55) | ( 1.11; 1.78) | ( 1.13; 1.81) | ( 1.11; 1.77) | ( 1.10; 2.46) | ( 1.09; 2.43) |
| **Otitis media** | 2.27 | 1.57 | 2.43 | 2.25 | 2.03 | 2.70 | 2.23 |
|  | ( 0.92; 5.65) | ( 0.67; 3.72) | ( 0.97; 6.11) | ( 0.91; 5.58) | ( 0.85; 4.81) | ( 0.62;11.72) | ( 0.51; 9.83) |
| **Pharyngitis/Laryngitis** | 1.64 | 1.34 | 1.74 | 1.71 | 1.62 | 1.52 | 1.50 |
|  | ( 1.19; 2.27) | ( 0.97; 1.85) | ( 1.25; 2.40) | ( 1.24; 2.35) | ( 1.18; 2.23) | ( 0.91; 2.54) | ( 0.89; 2.52) |
| **Tonsillitis** | 1.30 | 1.13 | 1.45 | 1.36 | 1.49 | 1.39 | 1.28 |
|  | ( 0.63; 2.70) | ( 0.53; 2.39) | ( 0.70; 2.99) | ( 0.67; 2.79) | ( 0.74; 3.01) | ( 0.41; 4.76) | ( 0.37; 4.38) |
| **Influenza-like illness** | 1.54 | 1.32 | 1.57 | 1.58 | 1.53 | 1.79 | 1.81 |
|  | ( 1.20; 1.98) | ( 1.03; 1.70) | ( 1.22; 2.02) | ( 1.23; 2.02) | ( 1.19; 1.96) | ( 1.20; 2.67) | ( 1.21; 2.70) |
| **Bronchitis** | 2.17 | 1.52 | 2.25 | 2.41 | 2.18 | 3.22 | 3.27 |
|  | ( 1.45; 3.24) | ( 1.01; 2.29) | ( 1.49; 3.41) | ( 1.60; 3.63) | ( 1.45; 3.29) | ( 1.72; 6.03) | ( 1.74; 6.15) |
| **Pneumonia** | 5.90 | 3.73 | 6.60 | 6.31 | 4.95 | 8.85 | 8.44 |
|  | ( 1.52;22.91) | ( 0.92;15.14) | ( 1.47;29.70) | ( 1.42;28.07) | ( 1.11;22.17) | ( 0.63;124.49) | ( 0.62;115.75) |
| **other acute resp. infections** | 0.81 | 0.64 | 0.77 | 0.80 | 0.76 | 1.04 | 1.00 |
|  | ( 0.42; 1.56) | ( 0.32; 1.26) | ( 0.39; 1.51) | ( 0.41; 1.58) | ( 0.39; 1.49) | ( 0.44; 2.43) | ( 0.43; 2.33) |
| **≥3 RTIs** | 2.05 | 1.54 | 2.16 | 2.15 | 2.01 | 2.60 | 2.61 |
|  | ( 1.45; 2.89) | ( 1.10; 2.17) | ( 1.52; 3.06) | ( 1.52; 3.04) | ( 1.43; 2.83) | ( 1.53; 4.40) | ( 1.54; 4.43) |
| **Long RTIs** | 2.31 | 1.75 | 2.29 | 2.44 | 2.28 | 2.86 | 2.73 |
|  | ( 1.65; 3.24) | ( 1.25; 2.45) | ( 1.62; 3.23) | ( 1.73; 3.43) | ( 1.63; 3.20) | ( 1.67; 4.88) | ( 1.60; 4.67) |
| **Upper 10% in diary score** | 2.13 | 1.53 | 2.13 | 2.23 | 2.08 | 2.93 | 2.86 |
|  | ( 1.52; 2.99) | ( 1.10; 2.14) | ( 1.51; 3.02) | ( 1.58; 3.14) | ( 1.48; 2.92) | ( 1.72; 4.98) | ( 1.68; 4.86) |
| **Seasonal level** |  |  |  |  |  |  |  |
| **≥4 months RTIs** | 2.43 | 1.84 | 2.39 | 2.71 | 2.46 | 3.44 | 3.37 |
|  | ( 1.48; 3.97) | ( 1.12; 3.03) | ( 1.44; 3.97) | ( 1.64; 4.49) | ( 1.50; 4.06) | ( 1.57; 7.53) | ( 1.54; 7.37) |
| **≥3 long RTIs** | 3.10 | 2.27 | 3.09 | 3.16 | 2.94 | 3.11 | 2.97 |
|  | ( 2.00; 4.81) | ( 1.47; 3.52) | ( 1.97; 4.84) | ( 2.03; 4.91) | ( 1.90; 4.57) | ( 1.65; 5.86) | ( 1.58; 5.59) |
| **Upper 10% in diary score** | 4.49 | 2.47 | 4.61 | 4.90 | 4.43 | 4.10 | 3.96 |
|  | ( 2.38; 8.49) | ( 1.33; 4.61) | ( 2.38; 8.92) | ( 2.55; 9.42) | ( 2.31; 8.46) | ( 1.85; 9.08) | ( 1.79; 8.78) |
| **Individual level** |  |  |  |  |  |  |  |
| **Upper 10% in diary score** | 2.22 | 1.48 | 2.17 | 2.33 | 2.17 | 3.15 | 3.05 |
|  | ( 1.44; 3.43) | ( 0.93; 2.36) | ( 1.42; 3.34) | ( 1.53; 3.55) | ( 1.42; 3.32) | ( 1.59; 6.24) | ( 1.54; 6.07) |

^a^ to allow for a comprehensive identification of biomarkers and other features of people susceptible to RTI, AWIS participants were invited into the study center for a detailed interview, anthropometric measurements, lung function test, pulse oximetry, a general medical examination as well as the collection of biosamples (blood, urine, oral and nasal swabs) and measurement of selected biomarkers including serum vitamin D levels. The recruitment started in August 2014 and it is still ongoing. From 1455 invited AWIS participants (546 male and 903 female), 550 (200 male and 348 female) were recruited until the end of January 2016. 508 of these (186 male and 322 female) with available BMI were included in the present analysis

b Adjustment was performed separately for each factor. Gender, education, smoking, contact to small children, asthma, co-morbidity score, score of removed organs, were added as categorical covariates; age groups, sport activity score, nutrition score, serum vitamin D were added as continuous covariates.
